# Evaluation of heritability partitioning approaches in livestock populations

**DOI:** 10.1186/s12864-024-10600-y

**Published:** 2024-07-13

**Authors:** Can Yuan, José Luis Gualdrón Duarte, Haruko Takeda, Michel Georges, Tom Druet

**Affiliations:** 1 Unit of Animal Genomics, GIGA-R & Faculty of Veterinary Medicine, University of Liège, Avenue de L’Hôpital, 1, 4000 Liège, Belgium; 2 Walloon Breeders Association, Rue Des Champs Elysées, 4, Ciney, 5590 Belgium

**Keywords:** Heritability partitioning, Variance components, Genetic architecture, Functional annotation, Complex traits

## Abstract

**Background:**

Heritability partitioning approaches estimate the contribution of different functional classes, such as coding or regulatory variants, to the genetic variance. This information allows a better understanding of the genetic architecture of complex traits, including complex diseases, but can also help improve the accuracy of genomic selection in livestock species. However, methods have mainly been tested on human genomic data, whereas livestock populations have specific characteristics, such as high levels of relatedness, small effective population size or long-range levels of linkage disequilibrium.

**Results:**

Here, we used data from 14,762 cows, imputed at the whole-genome sequence level for 11,537,240 variants, to simulate traits in a typical livestock population and evaluate the accuracy of two state-of-the-art heritability partitioning methods, GREML and a Bayesian mixture model. In simulations where a single functional class had increased contribution to heritability, we observed that the estimators were unbiased but had low precision. When causal variants were enriched in variants with low (< 0.05) or high (> 0.20) minor allele frequency or low (below 1st quartile) or high (above 3rd quartile) linkage disequilibrium scores, it was necessary to partition the genetic variance into multiple classes defined on the basis of allele frequencies or LD scores to obtain unbiased results. When multiple functional classes had variable contributions to heritability, estimators showed higher levels of variation and confounding between certain categories was observed. In addition, estimators from small categories were particularly imprecise. However, the estimates and their ranking were still informative about the contribution of the classes. We also demonstrated that using methods that estimate the contribution of a single category at a time, a commonly used approach, results in an overestimation. Finally, we applied the methods to phenotypes for muscular development and height and estimated that, on average, variants in open chromatin regions had a higher contribution to the genetic variance (> 45%), while variants in coding regions had the strongest individual effects (> 25-fold enrichment on average). Conversely, variants in intergenic or intronic regions showed lower levels of enrichment (0.2 and 0.6-fold on average, respectively).

**Conclusions:**

Heritability partitioning approaches should be used cautiously in livestock populations, in particular for small categories. Two-component approaches that fit only one functional category at a time lead to biased estimators and should not be used.

**Supplementary Information:**

The online version contains supplementary material available at 10.1186/s12864-024-10600-y.

## Background

In livestock species, the number of genotyped and whole-genome sequenced animals is steadily increasing. Combining these data with missing genotype imputation techniques allows genome-wide association studies and genomic selection to be performed at the sequence level in large cohorts. More recently, functional annotations of the genome are becoming available for several livestock species [1, 2]. For example, transcriptome data [3, 4], chromatin accessibility maps [5, 6] or histone mark distributions [6, 7] are now available in cattle. In human genetics, such information has been used to study the genetic architecture of complex traits, including complex diseases [8, 9]. More precisely, the contribution of different functional categories of variants to the genetic variance of these different traits has been estimated. Such approaches are referred to as variance partitioning or heritability partitioning approaches. They have for example highlighted the importance of regulatory variants [8, 9]. Fewer studies have been realized in livestock species, as functional annotation maps remain limited compared to humans, and are more recent. Nevertheless, similar approaches have been used, for instance, in cattle [10, 11]. In this context, the identification of functional categories contributing to complex traits is also important for prioritizing variants to be used in genomic selection and improving its accuracy.

Most methods used for heritability partitioning have been developed and tested in the context of human genetics [9, 12, 13]. Although livestock species have specific characteristics at the genomic level, methods have often been transferred without additional testing. As a result of their demographic history, including domestication, breed creation and intensive selection, livestock species are indeed different in terms of effective population size [14, 15], levels and extent of linkage disequilibrium (LD) [16], relatedness between individuals and levels of inbreeding [17]. The higher selection intensity in livestock species often results in the fixation of large effect variants accompanied by large selective sweeps [18]. Importantly, previous studies in humans have relied on samples of unrelated individuals, discarding all pairs of individuals with a relatedness level above 0.025 [12], whereas in a typical livestock dataset these and higher relationships are common. For instance, with the use of artificial insemination, many individuals may have a common sire or grand-sire. Similarly, the importance of accounting for LD scores when estimating variance components [12] has not been evaluated when high LD levels are present at long distances [19].

We herein used a genotyped population of 14,762 Belgian Blue Beef (BBB) cows to evaluate the accuracy of heritability partitioning approaches in a typical livestock population. Belgian Blue cattle have indeed been intensively selected for muscular development. This has resulted in the fixation of an 11 bp deletion in the myostatin gene [20], accompanied by a large selective sweep [18]. Additional genetic variation for muscular development has been exploited to further improve this trait [21]. As in other livestock populations, the effective population size is small, around 100 [18], and individuals have high levels of recent inbreeding associated with long runs of homozygosity [22]. The objective of the present study was to use these data to perform realistic simulations, with characteristics of a typical livestock population, in order to evaluate two state-of-the-art methods, a variance component approach [12] and a Bayesian mixture model [13]. The simulations included scenarios where causal variants were enriched in specific allele frequency, LD score or functional categories. An additional objective was to use these approaches to perform heritability partitioning based on functional annotation for muscular development and height traits in Belgian Blue beef cattle.

## Methods

### Data

For the present study we used data from 14,762 Belgian Blue beef cows with imputed genotypes from 11,537,240 SNPs and small indels [23]. Cows were genotyped with either low-density (9983 to 20,502 SNPs) or medium-density (51,809 to 57,979 SNPs) arrays and genotype imputation to the sequence level was performed in successive steps. The reference panels included 13,600, 890 and 230 individuals at the medium-density (28,893 SNPs selected), high-density (572,667 SNPs selected) and sequence levels, respectively. Variants with low minor allele frequency (MAF) (< 0.01) or with lower imputation accuracy (r^2^ < 0.90) were filtered out, resulting in the selection of 11,431,742 variants. More details on the imputation procedure and the data set can be found in Gualdrón Duarte et al. [23]. We used phenotypes for muscularity traits (shoulder muscularity, top muscularity, buttock muscularity rear and side view) and height (with heritabilities of 0.30, 0.31, 0.42, 0.39 and 0.38, respectively). The four muscularity traits are scores from 51 to 100, given on the farm by a technician based on a visual assessment (available for 14,476 individuals), while height was measured for 12,904 individuals. In addition, a synthetic score for muscular development was obtained as a linear combination of the four individual muscularity scores (with a weight of 1 for shoulder and top muscularity and 2 for buttock muscularity scores). These phenotypes were corrected for fixed effects from the evaluation model as described in Gualdrón Duarte et al. [23].

### Variant annotation

For variant annotation, we selected categories similar to those defined by Gusev et al. [8]. Accordingly, six functional categories were defined to classify the 11,431,742 variants. First, we identified variants located in open chromatin regions (OCR). These regions were defined using an organism-wide catalog of 976,813 cis-acting regulatory elements for the bovine detected by the assay for transposase accessible chromatin using sequencing (ATAC-SEQ) described in Yuan et al. [5]. The catalogue was generated using data from 106 samples corresponding to 68 tissue types. We annotated as OCR variants those variants located in the 976,813 peaks, which represented 10% of the genome space. Variants outside the OCR were classified into five additional groups corresponding to coding sequence (CDS), untranslated regions (UTR) including both 3’ and 5’ UTR, regions upstream (-1 kb) or downstream (+ 1 kb) of genes (UDR), intronic (IOR) and intergenic (IGR) regions. The number of variants per category is reported in Table 1. This annotation was obtained from the General Transfer Format (GTF) file of the bovine genome assembly ARS-UCD1.2 downloaded from Ensembl (v105). This file directly provides coordinates of genes, transcripts, exons, CDS and UTR. IORs were defined as non-exonic regions in genes. Transcription start and termination sites (TSS and TTS) were obtained using Homer [24] and all transcripts from the genes. Upstream and downstream regions were then defined as 1 kb upstream and downstream from TSS and TTS, respectively. IGR corresponded to the remaining unannotated regions.

**Table 1:** Description of the number of variants in each functional category and their contribution to SNP heritability in the three more complex scenarios

Annotation	Full genome		Subset of the genome		%SNP heritability		
	Number of variants	Proportion of variants	Number of variants	Proportion of variants	Scenario I	Scenario II	Scenario III
CDS	63,663	0.56%	4,161	0.43%	25	15	50
IOR	3,445,739	30.14%	278,021	28.80%	14	10	20
UTR	5,837	0.05%	412	0.04%	1	0.5	0
UDR	604,425	5.29%	40,423	4.19%	10	4.5	10
IGR	6,099,183	53.35%	547,980	56.76%	15	10	0
OCR	1,212,895	10.61%	94,429	9.78%	35	60	20

*CDS* Coding sequence, *IOR* Intronic regions, *UTR* 5’ and 3’ untranslated regions, *UDR* Up- and down-stream regions, *IGR* Intergenic regions, *OCR* Open chromatin regions

Annotation groups were also defined based on MAF and linkage disequilibrium (LD) scores [12]. Three MAF groups were defined [0.01–0.05; 0.05–0.10; 0.10–0.50]. For each variant, LD scores were obtained using GCTA [25] as the sum of LD r^2^ scores between the variant and all variants within a 200 kb window [12]. SNPs were then stratified into four LD score groups based on quartiles. These groups thus represent SNPs that have, for example, low or high LD levels with other SNPs in the region. SNPs in high LD groups capture the effect of more SNPs, and potentially causal variants, than SNPs in low LD groups.

### Heritability partitioning methods

Two methods were applied to estimate the contribution of different annotation groups to the additive genetic variance. First, we used a genomic restricted maximum likelihood (GREML) approach to estimate the variance components with the following linear mixed model:

$${\varvec{y}}=1\mu +\sum_{s=1}^{S}{{\varvec{g}}}_{s}+{\varvec{e}}$$

where ***y*** is the vector of individual phenotypes, $1\mu$ is the intercept term (i.e. the mean effect), ***g***_s_ is the vector of individual polygenic effects associated to annotation group *s*, *S* is the total number of fitted annotation groups, and ***e*** is the vector of individual random error terms. Each polygenic component is normally distributed, ${{\varvec{g}}}_{s} \sim N(0,{{{\varvec{G}}}_{s}\sigma }_{s}^{2})$ where ***G***_s_ is the genomic relationship matrix (GRM) computed using the variants present in category *s* and ${\sigma }_{s}^{2}$ is the variance of polygenic effects from the annotation group. The GRM were computed with GCTA using centered and scaled genotypes as described in Yang et al. [25]. The residual error terms are independent and normally distributed, ${\varvec{e}} \sim N(0,{{\varvec{I}}\sigma }_{e}^{2})$ where ***I*** is the identity matrix and ${\sigma }_{e}^{2}$ is the residual variance. The additive polygenic variance, ${\sigma }_{g}^{2}$, is equal to the sum of the variances associated to each annotation groups:

$${\sigma }_{g}^{2}=\sum_{s=1}^{S}{\sigma }_{s}^{2}$$

The contribution of annotation group *s* to the genetic variance, called %SNP heritability, is estimated as:

$${\%h}_{s}^{2}=\frac{{\sigma }_{s}^{2}}{{\sigma }_{g}^{2}}$$

Variance components were estimated using GCTA and the Average-Information (AI) algorithm (default option). When the AI-REML did not converge, we used the EM-REML algorithm with a maximum of 500 iterations.

The second approach is a Bayesian model designed for large-scale genomic data and called BayesRR-RC [13]. The model is an extension of BayesR [26] and BayesRC [27]. Variant effects are described as a mixture of null effects (spike probability at zero) and Gaussian distributions. The hyper-parameters vary for variants from different annotation groups. Accordingly, the variance explained by the markers and their mixture proportions are group-specific. Phenotypes are modeled as:

$${\varvec{y}}=1\mu +\sum_{s=1}^{S}{{\varvec{X}}}_{s}{{\varvec{\beta}}}_{s}+{\varvec{e}}$$

where ***X***_s_ is the matrix of centered and scaled genotypes for markers in category s and ***β***_s_ is the vector of marker effect for category *s*. These effects are distributed according to:

$${{\varvec{\beta}}}_{{s}_{j}}\sim {\pi }_{{0}_{s}}{\delta }_{0}+{\pi }_{{1}_{s}}N(0,{\sigma }_{{1}_{s}}^{2})+{\pi }_{{2}_{s}}N(0,{\sigma }_{{2}_{s}}^{2})+{\dots +\pi }_{{L}_{s}}N(0,{\sigma }_{{L}_{s}}^{2})$$

where *j* is the marker index, δ_0_ is a discrete probability mass at 0, *L* is the number of Gaussian distributions in the mixture, $\left\{{\pi }_{{0}_{s}},{\pi }_{{1}_{s}}, {\pi }_{{2}_{s}}, \dots ,{\pi }_{{L}_{s}}\right\}$ are the mixture proportions for annotation group *s*, $\left\{{\sigma }_{{1}_{s}}^{2},{\sigma }_{{2}_{s}}^{2}, \dots ,{\sigma }_{{L}_{s}}^{2}\right\}$ are the mixture variances for group *s*, proportional to ${\sigma }_{s}^{2}$, the variance explained by the group which is directly estimated from the data. In our study, we set *L* to 3, with variances ${\sigma }_{{l}_{s}}^{2}$ respectively equal to 0.0001, 0.001 and 0.01 ${\sigma }_{s}^{2}$. This model was run using the GMRM software [13] with a Gibbs sampling scheme for 5,000 iterations with a burn-in period of 2,000 iterations. This setting corresponds to the values used by the software developers in their original study [13], and Orliac et al. [28] have shown that 2,000 iterations allow to obtain good approximations of the parameters.

Different definitions of annotation groups can be applied in both approaches. In two-component (TC) models, two functional annotation groups are selected (e.g., OCR versus non-OCR), whereas in multiple-component (MC) models, multiple functional annotation groups are fitted simultaneously. Additional stratification levels can be added to these models [12]. In the MAF-stratified (MS) and LD-stratified (LDS) models, groups are defined as a function of the MAF and LD score categories described above, respectively, whereas an LDMS model fits all combinations of functional, MAF and LD categories. In this case, the total number of fitted components is equal to the number of functional categories multiplied by the number of MAF groups and by the number of LD score groups. When a model is run without correcting for MAF or LD score categories, we use the abbreviation "noLDMS" to distinguish it from the other models.

### Simulation study

#### Phenotype simulation

To obtain phenotypes with different architectures, we simulated them as:

$${\varvec{y}}=\sum_{s=1}^{S}\sum_{j=1}^{{M}_{s}}{{\varvec{x}}}_{{s}_{j}}{\beta }_{{s}_{j}}+{\varvec{e}}$$

where **y** is the vector of individual simulated phenotypes, *S* is the number of different annotation groups, *M*_s_ is the number of causal variants (CVs) in annotation group *s*, ***x***_*sj*_ is a vector of centered individual allele dosages for the *j*^th^ variant from the *s*^th^ group, β_*sj*_ is the effect of the corresponding variant and ***e*** is a vector of individual errors terms. By default, CV effect sizes were sampled from normal distributions with variance equal to [2*p*_*j*_(1-*p*_*j*_)]^−1^, where *p*_*j*_ is the allele frequency of variant *j*. This is equivalent to assuming that each CV contributes equally to the genetic variance, as in Gusev et al. [8] and Yang et al. [12]. This corresponds also to the default rule used by GCTA to construct the GRM. We assessed the robustness to this assumption later (see below). To simulate variable contributions of the annotation groups to the genetic variance, we selected the number of CVs, M_s_, proportionally to the simulated contribution.

In this model, the individual polygenic effects *g*_i_ are equal to:

$${\varvec{g}}=\sum_{s=1}^{S}\sum_{j=1}^{{M}_{s}}{{\varvec{x}}}_{{s}_{j}}{\beta }_{{s}_{j}}=\sum_{s=1}^{S}{{\varvec{g}}}_{s}$$

where ***g*** is the vector of individual polygenic effects and ***g***_s_ is the vector of individual polygenic effects associated to annotation group *s*. After simulating these polygenic terms, their variance was rescaled to obtain the simulated contribution to the genetic variance, also defined as %SNP heritability. Finally, the individual error terms were normally distributed with a variance adjusted to obtain the simulated heritability. By default, *M*, the total number of CVs, was set equal to 10,000 and the heritability to 0.50. This simulation code is available at https://github.com/can11sichuan/Bov-hg/.

#### Simulations scenarios with causal variants enriched in OCR

In unstratified scenarios, CVs were randomly sampled. In other scenarios, higher proportions of variants were sampled in certain annotation groups.

We started with simulations in which OCR contributed to 50% of the heritability, without stratification according to MAF or LD scores. Accordingly, 5,000 CVs were selected within OCR and 5,000 outside OCR. We then ran simulations in which CVs were enriched in specific MAF classes, LD-score categories, or combinations of both (LDMS simulation scenarios). The enriched annotation groups were defined as low MAF (MAF ≤ 0.05), high MAF (MAF > 0.20), low LD (LD scores below the 1st quartile) and high LD (LD scores above the 3rd quartile). In these simulations, 3,000 OCR SNPs were sampled in the enriched annotation groups and 2,000 OCR SNPs were sampled outside of these groups, and the same sampling was applied outside of OCR. A total of six stratified scenarios were defined: 1) low MAF, 2) high MAF, 3) low LD, 4) high LD, 5) low MAF and low LD, and 6) low MAF and high LD.

Finally, we tested the robustness of the approaches to the relationship between SNP effects and their MAF. In the default scenario described above, CVs have the same contribution to the genetic variance (i.e. rare variants have larger effects). In the alternative scenario corresponding to the first rules proposed by VanRaden [29], the distribution of CV effects was independent of MAF (common variants would have a higher contribution to genetic variance).

Due to the high computational demands of BayesRR-RC, we worked with a subset of the genome. To do this, we randomly sampled 200 positions in the genome and selected all variants within 500 kb of the position (we sampled fragments rather than variants to preserve some LD structure). This resulted in a selection of 191 Mb and 965,428 variants (we have less than 200 Mb because some positions were less than 500 kb apart and their windows overlapped, while other positions were close to the chromosome ends). Both BayesRR-RC and GREML were applied to these simulations to ensure fair comparisons.

In total, each simulation scenario was repeated 100 times.

#### Simulation scenarios with variable contributions from different functional categories

We then used the six functional categories in our simulations. These categories were similar to those used in the study by Gusev et al. [8]. As in their study, we ran simulations where one of the functional categories contributed to 100% of the genetic variance, and then simulations without enrichment, where each category contributed proportionally to the number of variants present in the category. In addition, we simulated three more complex scenarios in which the different functional categories had variable contributions (Table 1). For these simulations, repeated 100 times per scenario, the heritability was set to 0.70 and we selected 2,000 CVs variants. In the scenarios where a single class contributed to 100% of the heritability, the number of CVs was reduced to 500, as the number of SNPs in certain categories was limited.

#### Evaluation metrics

For each scenario, we reported summary statistics (mean, median, standard deviation, quantiles, minimum, maximum), measures of precision and accuracy (Root Mean Square Error – RMSE, and bias) of the estimators. We also reported the number of simulations without convergence with the AI-REML and after 500 additional iterations of the EM-REML.

#### Application to real data

Finally, we applied the approach to the five muscular development traits and height measured on the ~ 15,000 genotyped Belgian Blue beef cows and imputed to the whole-genome sequence level. We used a MC model with the same partitioning of the genome as in the simulation, except that UTR was merged with CDS (as the variability of estimates in the small category was too high). For the GREML approach, GRMs were computed using the rules described above [25] or the first rule proposed by VanRaden et al. [29]. In addition, we also estimated the %SNP heritability associated with the different annotation classes using a TC approach.

## Results

### Estimation of proportion of genetic variance associated with a single annotation class

**Fig. 1:**
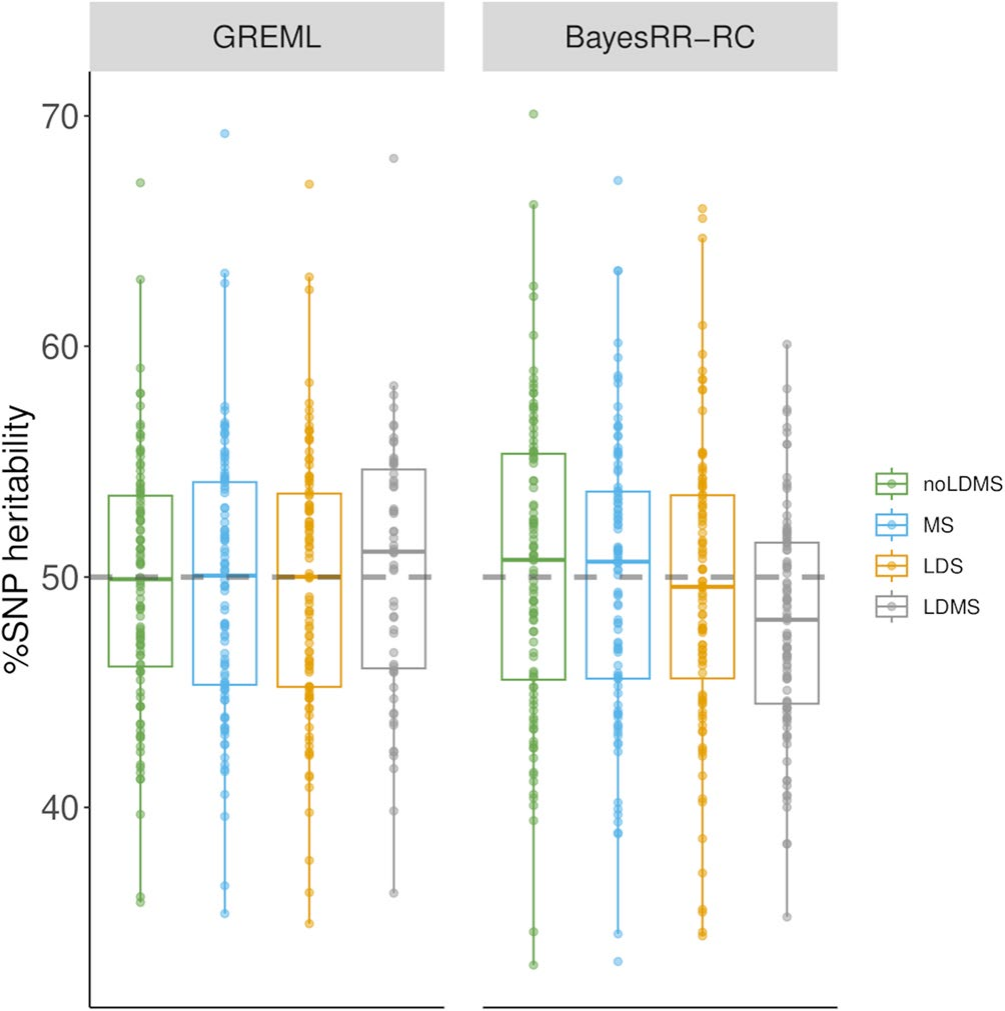
Estimation of %SNP heritability when variants in open chromatin regions (OCR) accounted for 50% of heritability. There was no additional MAF (MS) or LD stratification (LDS) in the simulations. The %SNP heritability was estimated with GREML and BayesRR-RC. The methods were applied without correction for MAF or LD score (noLDMS), and with MAF stratified (MS), LD stratified (LDMS) and both MAF and LD stratified (LDMS) approaches

We first assessed whether the approaches could estimate the proportion of genetic variance associated with a specific category (also referred to as %SNP heritability) with TC models. For this purpose, we selected variants located in open chromatin regions (OCR) identified by ATAC-SEQ [5], which account for approximately 10% of the genome, and started with simulations in which these variants accounted for 50% of the genetic variance. The architecture was independent of both MAF and LD scores (i.e. CVs were randomly sampled within OCR and non-OCR). In Fig. 1, we show the proportion of genetic variance estimated with GREML or with the BayesRR-RC model (without correction for LDMS (noLDMS), MAF-stratified (MS), LD-stratified (LDS) or LD- and MAF-stratified (LDMS) approaches). Results for each scenario are provided in Additional File 1, including summary statistics, measures of precision and accuracy, and convergence information.

We observed that the %SNP heritability associated with OCR was accurately estimated with the different GREML approaches (mean = 49.7% (noLDMS), 49.7% (MS), 49.7% (LDS) and 50.4% (LDMS)), although with relatively high imprecision of the estimators (RMSE = 5.4 (noLDMS), 5.7 (MS), 5.7 (LDS) and 5.6 (LDMS)) (Additional file 1: Table S1). For example, the estimated %SNP heritability ranged from 35.9 to 67.1% when running GREML without correction for LDMS (95% of the values were between 40.4 and 58.5%). BayesRR-RC also produced estimates close to the simulated values, but with slightly higher levels of variation than GREML with the noLDMS, MS and LDS approaches. In this first scenario, the bias was below 1% with both methods, except with BayesRR-RC for the LDMS approach.

**Fig. 2:**
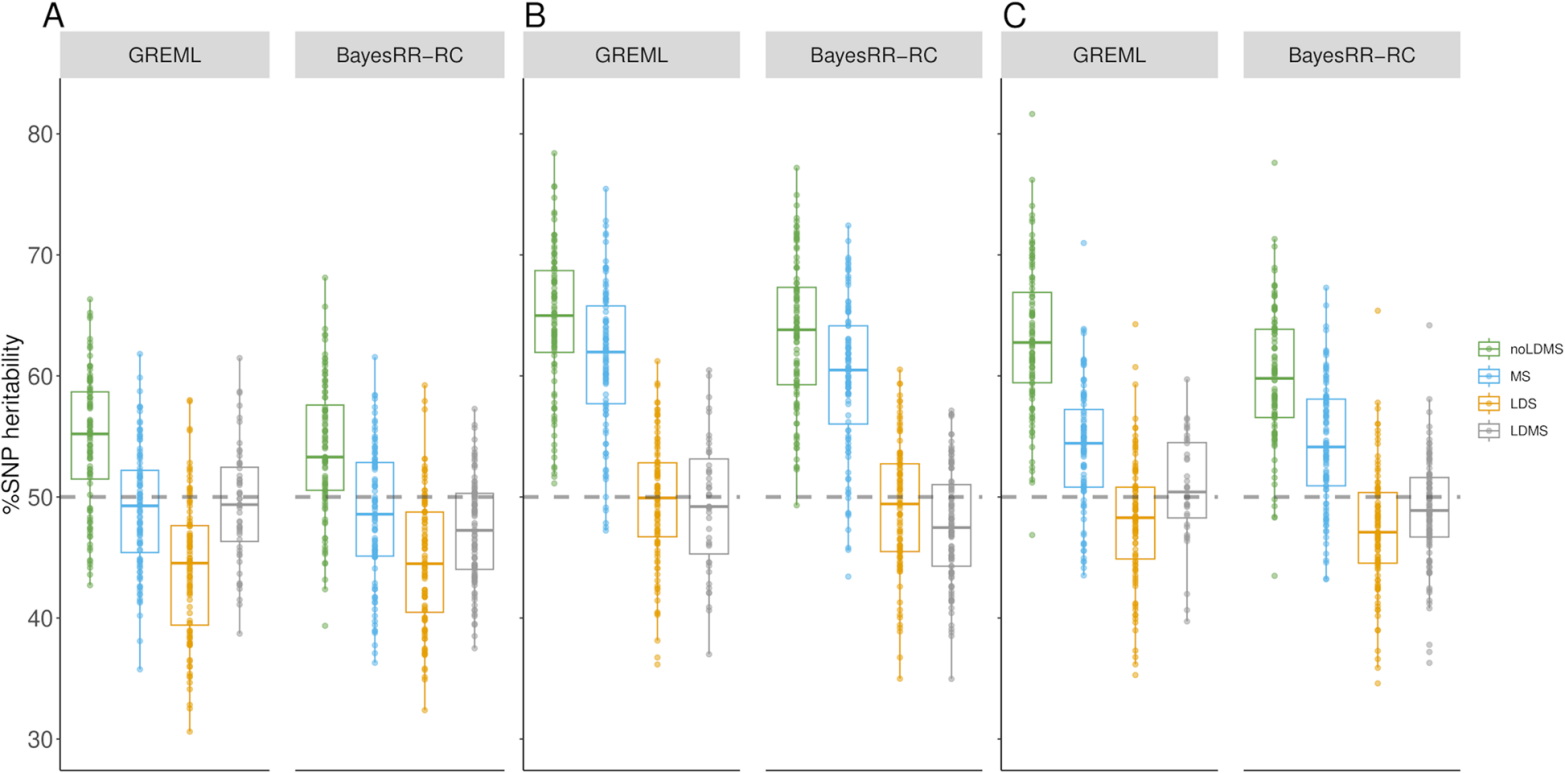
Estimation of %SNP heritability when causal variants are enriched in specific MAF or LD score categories. Variants in open chromatin regions (OCR) accounted for 50% of heritability. Causal variants were enriched in **A**) low MAF variants (MAF < 0.05), **B**) Low LD variants (LD score in the 1st quartile), and **C**) low MAF and low LD variants. The %SNP heritability was estimated with GREML and BayesRR-RC. The methods were applied without correction for MAF or LD score (noLDMS), and with MAF stratified (MS), LD stratified (LDMS) and both MAF and LD stratified (LDMS) approaches

Next, we investigated whether the methods were robust to MAF- or LD-dependent architectures (MS and LDS simulations, respectively). To this end, we performed simulations in which CVs were enriched in specific MAF classes (e.g., MAF ≤ 0.05 or MAF > 0.20), LD-score categories (i.e., SNP with LD score in the lower or upper quartile), or in combinations of both features (LDMS simulation scenarios). Although the noLDMS-GREML approach provided unbiased estimates of OCR %SNP heritability in some scenarios, such as the low MAF (Fig. 2A) and high MAF (Additional file 2: Figure S1A) scenario, high levels of bias were observed when CVs were enriched in certain LD classes (Fig. 2B-C and Additional file 2: Figure S1B). LDS-GREML was biased in MS simulations and vice versa. Overall, only LDMS models were robust in most scenarios (Fig. 2A-C; Additional File 1: Tables S2-7; Additional file 2: Figure S1), in agreement with previous studies [12, 30]. In this case, the estimators obtained with BayesRR-RC deviated more from the simulated values than the GREML approach. However, convergence was not systematically achieved with the GREML approach (with both the AI-REML algorithm and after 500 iterations of the EM-REML algorithm). This occurred mainly with the LDMS-GREML (Additional file 1: Tables S1-7), when a higher number of GRMs was fitted, and has also been reported in previous studies [9, 31].

In these first simulations, each CV had the same expected contribution to the genetic variance because its effect variance was proportional to the inverse of *p*_*j*_ (1- *p*_*j*_) (where *p*_*j*_ is the reference allele frequency at SNP *j*). This architecture is consistent with the default rule used to construct the GRM in GCTA (i.e., the same architecture was used in the simulation and in the partitioning approach). We also investigated whether the accuracy of heritability partitioning would be different if different rules were used to simulate the CV effects and to construct the GRMs used in the partitioning approach. Therefore, we performed the partitioning using GRMs constructed with the first rules proposed by VanRaden [29], assuming that the CV effect variance is independent of allele frequency. In addition, we used these second rules to simulate a new scenario in which common variants contribute more to the genetic variance. In the analyses, we observed a modest bias with the noLDMS and LDS approaches when the rules used to estimate the GRM did not match those used in the simulation (Additional file 2: Figure S2). Interestingly, this bias could be reduced by using the MS and LDMS approaches.

### Estimation of proportions of the genetic variance associated with multiple annotation classes

**Fig. 3:**
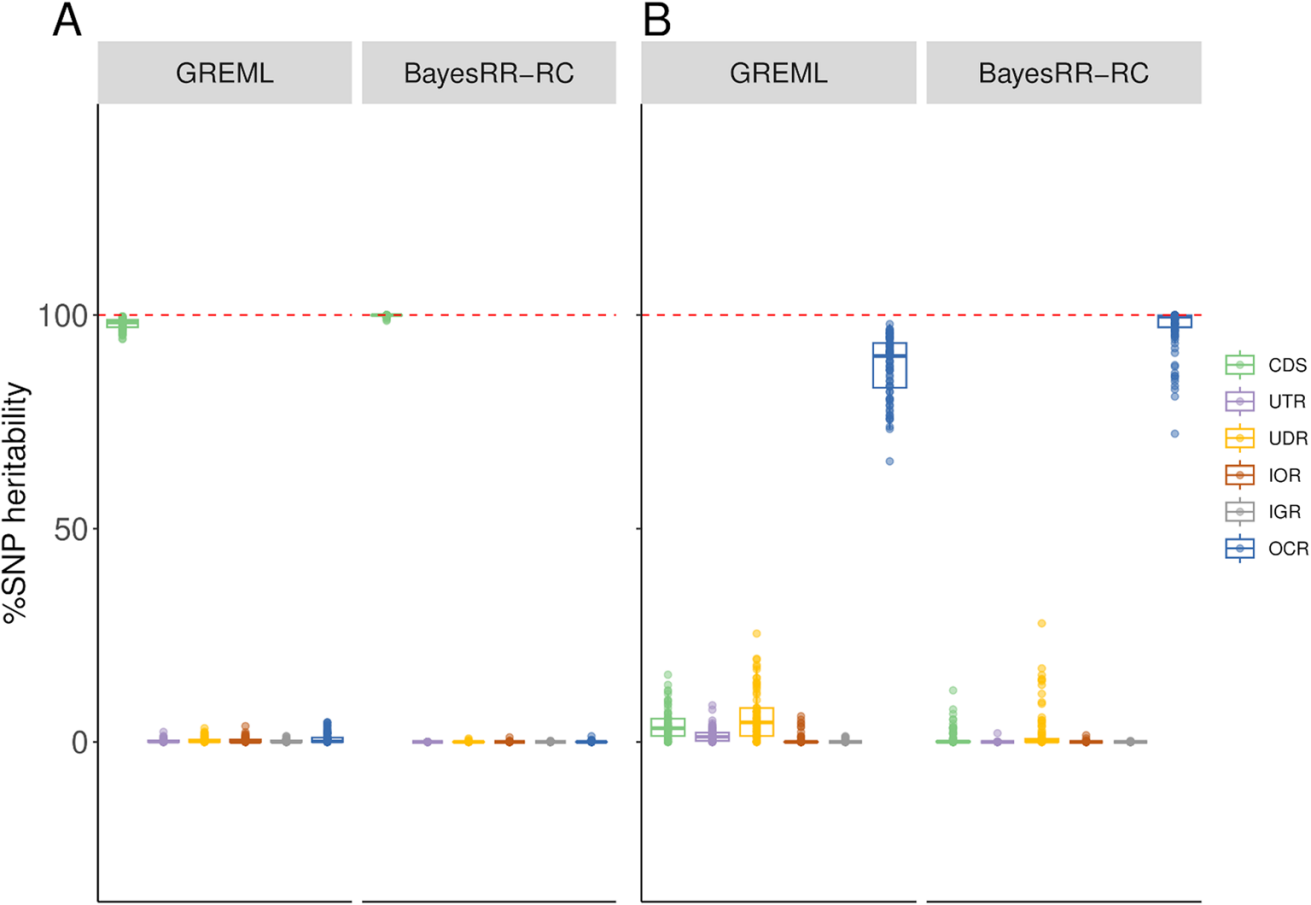
Estimation of %SNP heritability when causal variants are enriched in a single functional annotation class. Causal variants were located in **A**) coding sequences (CDS) and **B**) open chromatin regions (OCR). The %SNP heritability was estimated using GREML and BayesRR-RC with the following functional classes: CDS, 3’ and 5’ UTRs (UTR), upstream and downstream regions (UDR), intronic regions (IOR), intergenic regions (IGR) and OCR

**Fig. 4:**
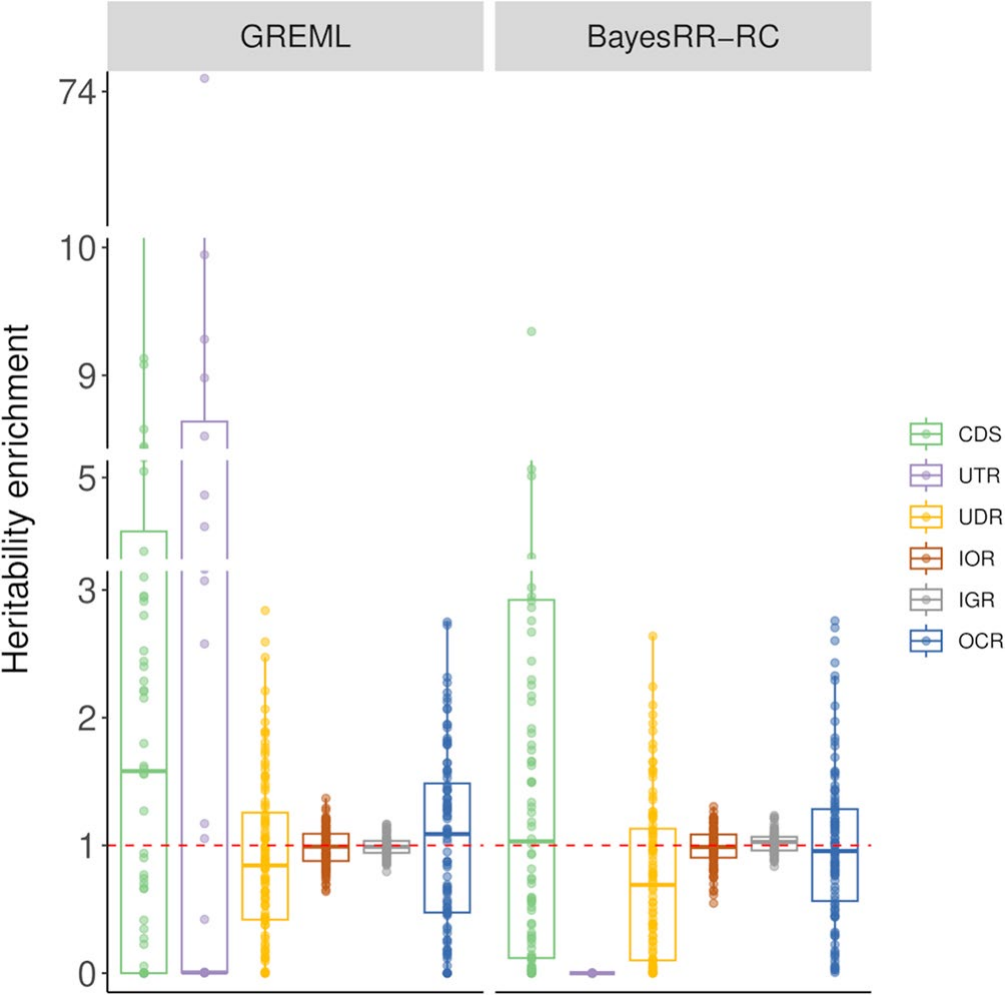
Estimation of heritability enrichment in simulations where SNPs from different functional classes had equal contribution. Heritability enrichment was estimated using GREML and BayesRR-RC with the following functional classes: coding sequence (CDS), 3’ and 5’ UTRs (UTR), upstream and downstream regions (UDR), intronic regions (IOR), intergenic regions (IGR) and open chromatin regions (OCR)

**Fig. 5:**
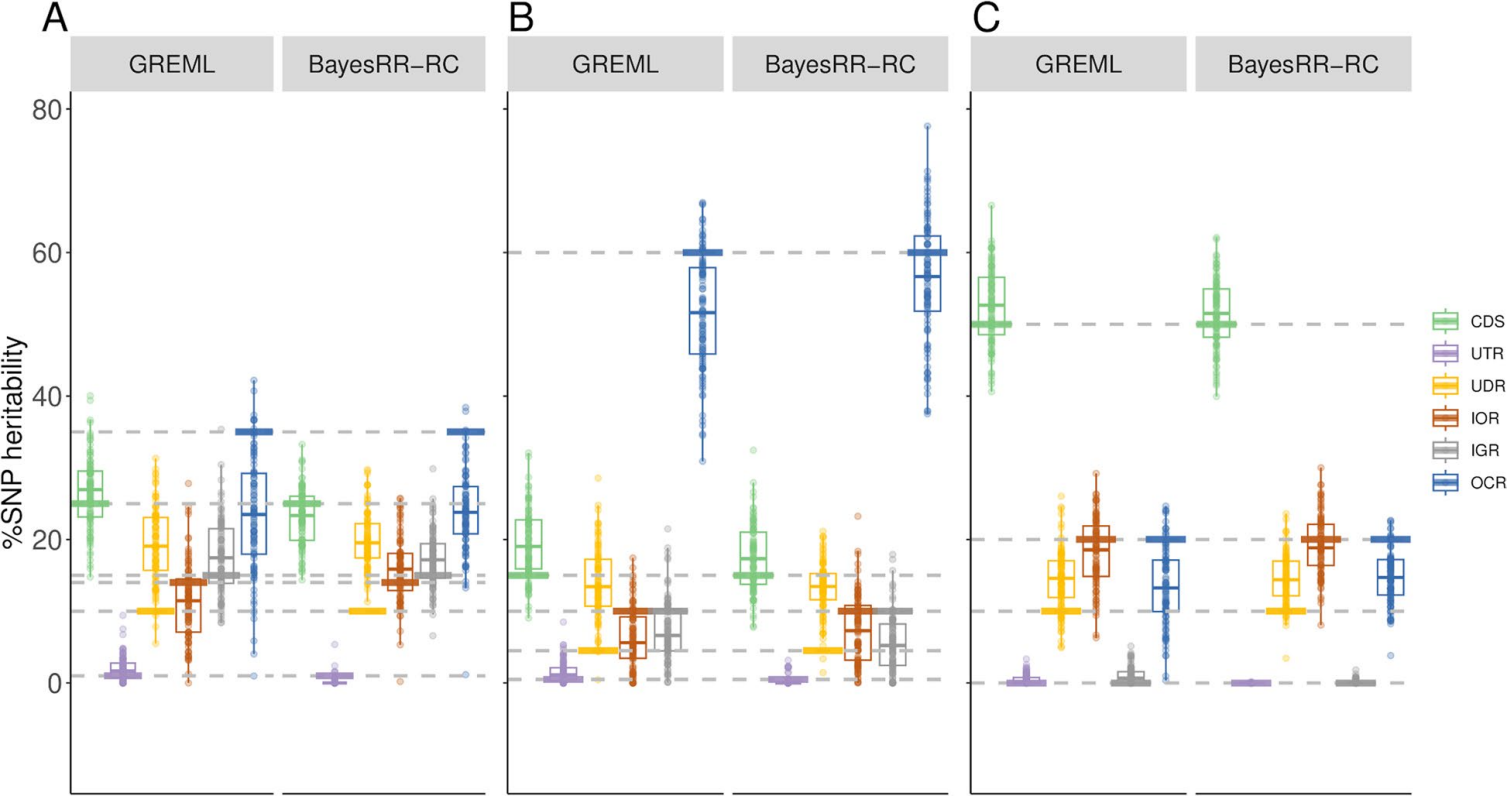
Estimation of %SNP heritability in complex simulation scenarios where SNPs from different functional classes had variable contributions. The contribution for each category is shown in Table 1. Heritability enrichment was estimated using GREML and BayesRR-RC with the following functional classes: coding sequence (CDS), 3’ and 5’ UTRs (UTR), upstream and downstream regions (UDR), intronic regions (IOR), intergenic regions (IGR) and open chromatin regions (OCR)

**Fig. 6:**
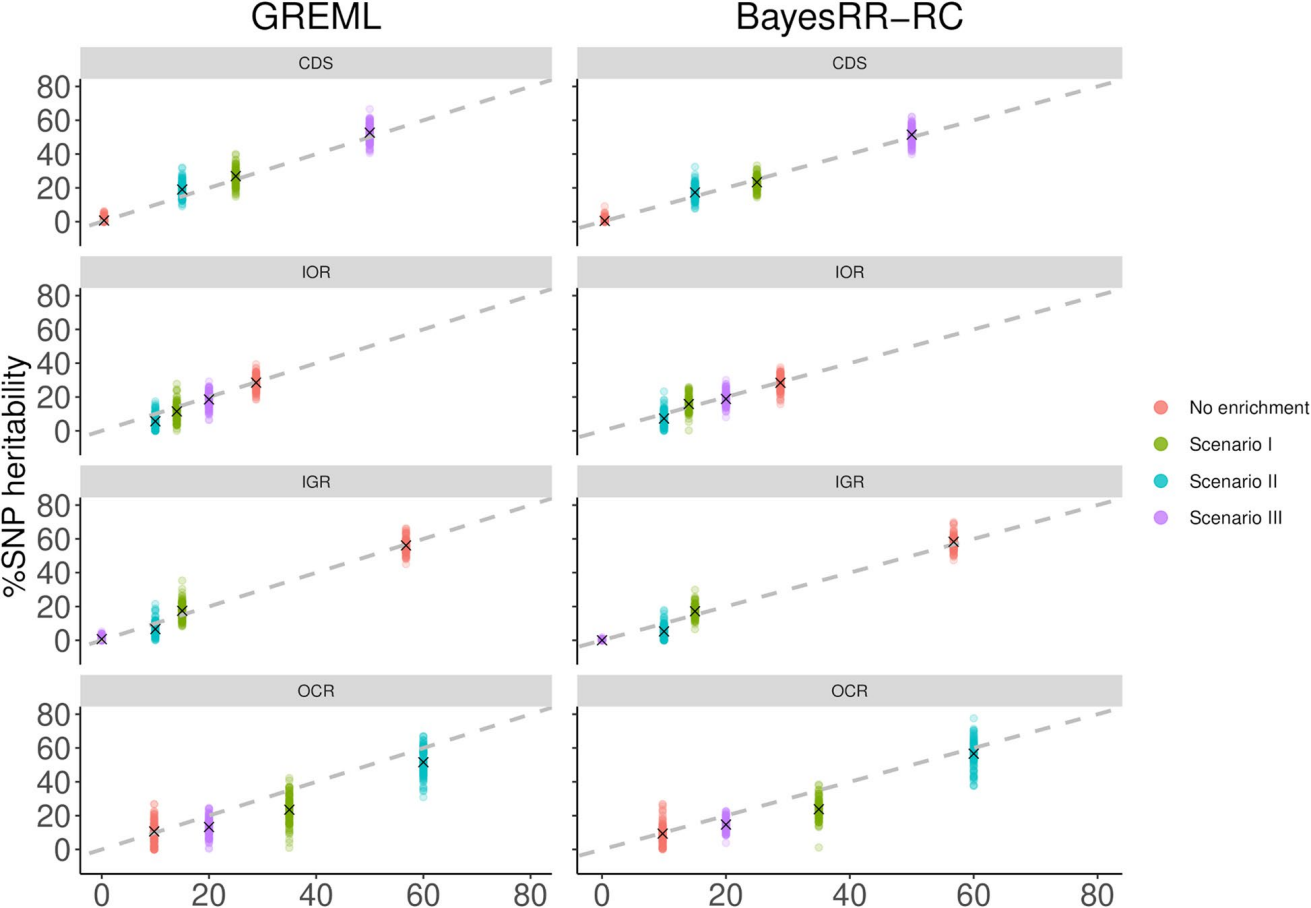
Scatterplot of estimated versus true %SNP heritability across simulation scenarios where SNPs from different functional classes contribute to genetic variance. The comparison is made separately for each functional class. %SNP heritability was estimated using GREML and BayesRR-RC with the following functional classes: coding sequence (CDS), 3’ and 5’ UTRs (UTR), upstream and downstream regions (UDR), intronic regions (IOR), intergenic regions (IGR) and open chromatin regions (OCR)

In the second part of the study, we simulated more complex scenarios in which six different annotation classes contributed to the total genetic variance to varying degrees. The selected categories were coding regions (CDS), 3’ and 5’ UTR (UTRs), regions upstream and downstream of genes (± 1 kb) called UDR, intronic regions (IOR), intergenic regions (IGR) and variants in OCR. For each simulation, we assessed whether the model was able to estimate %SNP heritability and heritability enrichment, defined as the ratio of the percentage of heritability contributed by the category to the percentage of SNPs in the category. To do this, we fitted the six categories simultaneously with a MC model, without correcting for LDMS structure for computational reasons. We started with simulations where all the genetic variance was associated to a single class (Fig. 3A-B; Additional file 1: Tables S8-12; Additional file 2: Figure S3). The GREML approach identified the class contributing to the genetic variation, but with relatively low precision and some bias (for instance, estimates ranged from 0.943 to 0.997 for CDS and from 0.657 to 0.979 for OCR). The BayesRR-RC approach was more accurate, with exceptionally low levels of variation in estimates across simulations, except when OCR variants accounted for 100% of the genetic variation. In this case, other categories such as CDS or UDR captured some of the variation, suggesting some confounding between these categories. We then ran simulations without heritability enrichment, with the proportion of CVs per category equal to their genomic proportions. For most classes, the correct levels of enrichment were estimated by both methods (Fig. 4; Additional file 1: Table S13), but some classes showed either high levels of variation or even some bias. The level of variation was inversely related to the size of the class, with the highest levels for the estimation of %SNP heritability for variants in UTRs and CDS. Overall, the %SNP heritability associated with each class and the ranking between classes was well estimated. We then simulated more complex and realistic scenarios with variable contributions from the different functional categories (see Table 1). In these scenarios, CDS and OCR were always enriched in causal variants, whereas intergenic and intronic regions harbored proportionally fewer causal variants. In the first scenario, five categories contributed 10% or more of the heritability, whereas OCR and CDS accounted for 50% or more of the genetic variation in the second and the third scenario, respectively. Results for the three scenarios are shown in Fig. 5A-C and Additional file 1: Tables S14-16. The standard deviations of the estimators were around 0.04, but higher values were observed for OCR (over 0.08). The estimators showed some bias, with deviations generally around 0.01–0.04. The largest biases were observed for OCR and UDR, which were underestimated and overestimated respectively, confirming the confounding between these categories. In most cases, the estimators obtained with BayesRR-RC were less variable and associated with lower biases. The average RMSE, combining variation and bias, was equal to 0.063 and 0.053 for GREML and BayesRR-RC, respectively (Additional file 1: Table S17). The ranking of the different categories according to their contribution to genetic variance was not always correct, with the largest errors associated with UDR, whose contribution was systematically overestimated, and OCR. Nevertheless, the estimators provided information about which classes contributed most to genetic variation (for example, the relative importance of CDS or intergenic variants was generally close to their simulated values). Comparisons of estimators from the same category across different scenarios (Fig. 6) indicate that these estimators are informative despite their low precision. The coefficient of determination from the regression of estimated versus simulated values was 0.941 for CDS, 0.760 for intronic regions, 0.959 for intergenic regions and 0.804 for OCR with GREML, and 0.947 for CDS, 0.708 for intronic regions, 0.965 for intergenic regions and 0.855 for OCR with BayesRR-RC. Note that for these analyses, we did not include scenarios where classes contribute to 100% of the genetic variance, and results for UDR are not shown because its simulated values remained low in all scenarios. We repeated this analysis using estimated heritability enrichment levels (Additional file 2: Figure S4).

**Fig. 7:**
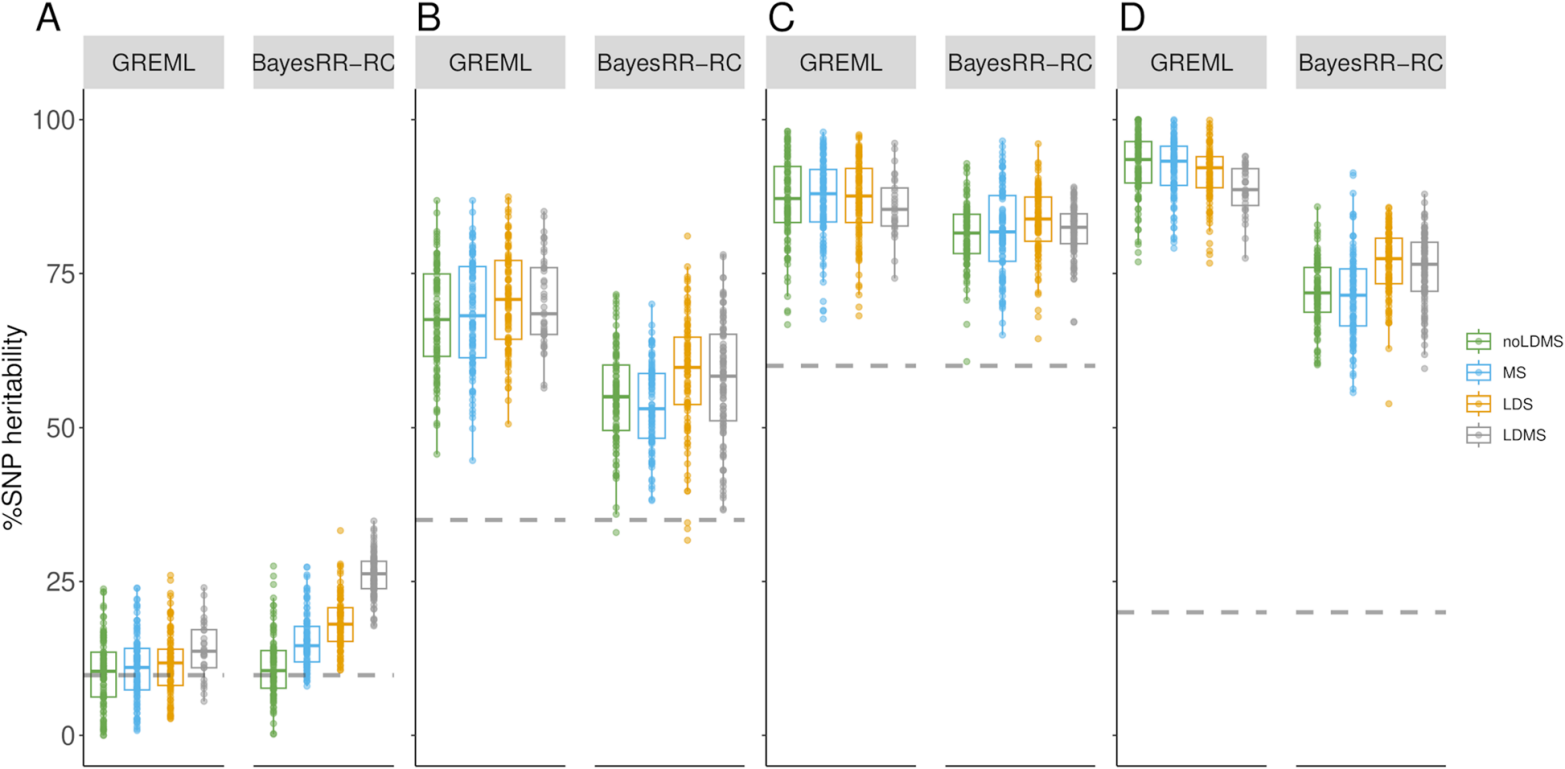
Estimation of %SNP heritability of variants in open chromatin regions (OCR) using a two-component strategy. Estimation was performed in complex simulation scenarios in which SNPs from multiple functional classes contribute to genetic variance (Panel **A** for the scenario without enrichment and Panels **B**-**D** for complex scenarios 1 to 3, respectively). Heritability enrichment was estimated using GREML and BayesRR-RC with the following two functional classes (OCR versus other categories). In addition, methods were run with unstratified (US), MAF stratified (MS), LD stratified (LDS) and both MAF and LD stratified (LDMS) approaches

We then evaluated the properties of the estimators obtained with models that estimate the contribution of only one functional category, using a model that fits a second category that includes all other functional classes (TC models). This approach is commonly used because it reduces computational requirements and thus allows MS, LDS or LDMS models to be applied. The approach was evaluated in the four scenarios where several categories contribute to the genetic variance, and not for UTR as the estimator was shown to be highly inaccurate due to the small size of the category. This strategy gave poor results as %SNP heritability was most often overestimated for all categories (OCR, UD, CDS, and IOR), even when LDMS methods were used, while biases were lower for intergenic regions (Fig. 7A-D; Additional file 1: Table S18-21; Additional file 2: Figure S5-8). The estimators showed no bias mainly in simulations without enrichment or when the category had a null contribution in the simulation. Bias was greater for OCR than for intergenic regions. In the vast majority of cases, heritability partitioning with multiple annotation groups gave better results, for example in terms of RMSE (Additional file 1: Table S22). This can also be observed when comparing estimates for a single category across multiple scenarios (Additional file 2: Figure S9). This behavior could occur because the fitted class captured variance associated with other classes due to their similarity (for example, in terms of GRM). We measured the correlations between the off-diagonal elements from GRM of each category (Additional file 1: Table S23) and observed, for example, that the GRM from IGR variants was less correlated with other GRMs, consistent with the fact that less confounding was obtained for this category. Other GRMs were highly correlated with the exception of the UTR GRM, probably because it was the smallest category. However, the correlation between GRMs from OCR and UDR was not the highest, even though they appeared to be the most confounded indicating that other parameters influence the confounding level. For example, relative distribution of effect sizes is probably important as we don’t observe confounding when enrichment levels are uniform across categories.

### Heritability partitioning for traits related to muscular development and height in cattle

**Fig. 8:**
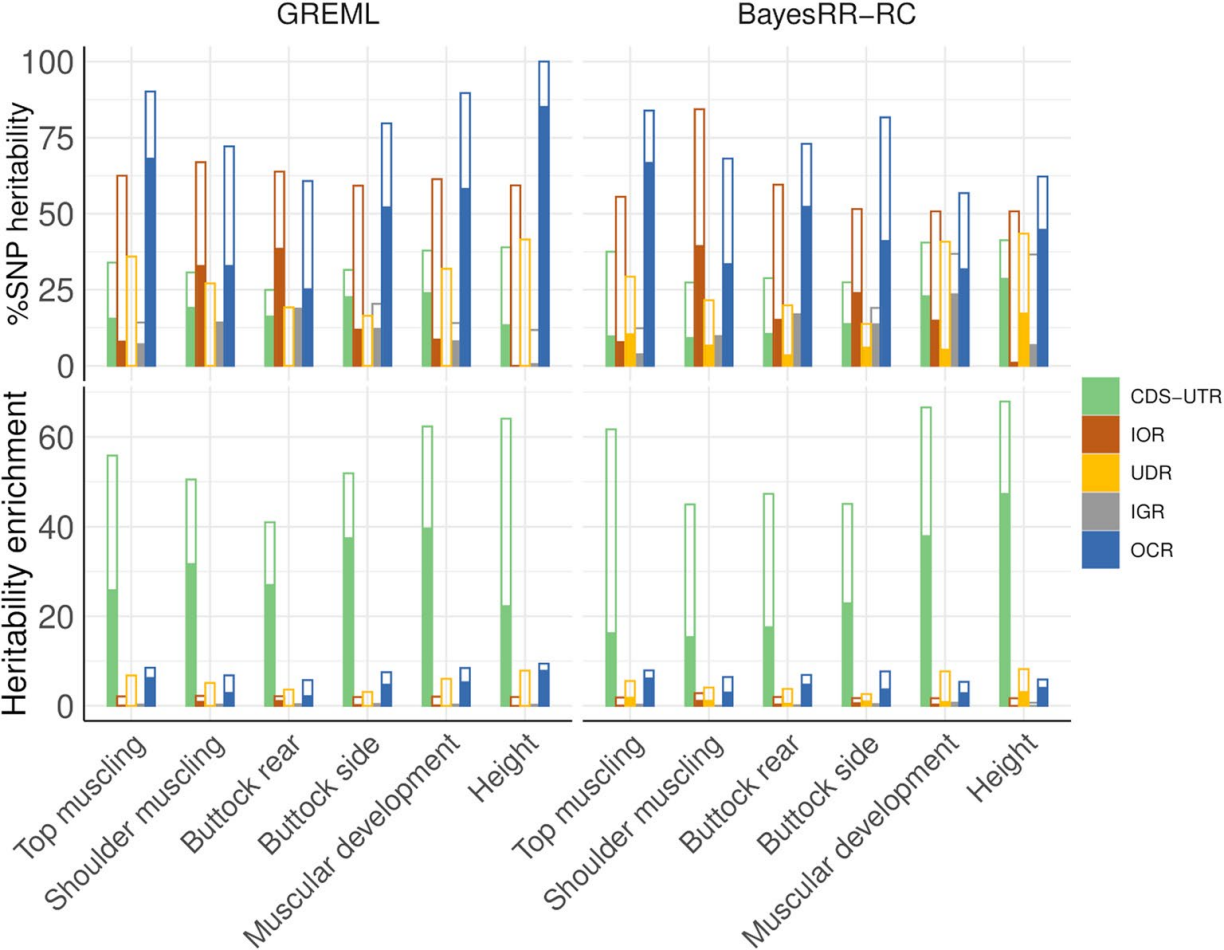
Estimation of %SNP heritability and heritability enrichment in real data sets. Estimates were obtained using GREML and BayesRR-RC with the following functional classes: coding sequence (CDS), 3’ and 5’ UTRs (UTR), upstream and downstream regions (UDR), intronic regions (IOR), intergenic regions (IGR) and open chromatin regions (OCR). Solid bars show %SNP heritability estimated when fitting simultaneously all the functional classes, while parameters estimated using a two-component approach, which only fits one functional category at a time, are shown with open bars

Finally, we applied the approach to the real phenotypes, as described in Material and methods. %SNP heritabilities from the different categories were relatively variable across traits. For instance, the contributions of intergenic or CDS-UTR variants estimated by BayesRR-RC were not consistent across traits, ranging from 10 to 30% of the genetic variation (Fig. 8; Additional file 1: Table S24). Similarly, relatively large differences were observed between BayesRR-RC and GREML estimates (for instance, the estimated %SNP heritability associated with OCR was equal to 85.5 and 45.0% for height). Nevertheless, some trends were consistent across traits and methods. OCR contributed to more than 30% of the genetic variance for all traits with BayesRR-RC (25% with GREML) and most often had the largest value of %SNP heritability (Fig. 8). The contribution of UDR was generally low, while intergenic variants had a modest contribution despite accounting for more than 50% of SNPs and indels. As in other studies, we averaged the contributions across traits [8, 9] (Table 2). For CDS-UTR, OCR, IGR and IOR, the average estimated contributions were similar with GREML and BayesRR-RC: over 45% for OCR, around 16–19% for CDS-UTR, 17% for IOR and 10–13% for IGR. UDR had a small contribution with both approaches, but almost zero with GREML (indicating possible problems in estimating the contribution of UDR with GREML). Except for CDS-UTR, the relative ranking of the different functional categories were consistent with both methods. In terms of heritability enrichment, some trends were also consistent (Fig. 8; Table 2; Additional file 1: Table S25). CDS-UTR had the largest enrichment (around 25 to 30-fold), followed by OCR (around fivefold on average), whereas intronic and intergenic variants had values below 1 (0.6-fold and 0.2-fold, respectively). Partitioning with a GREML using GRMs computed with the first rules proposed by VanRaden [29] was relatively similar to the first GREML results (Table 2; Additional file 1: Tables S24-25). The estimated contributions to heritability of CDS-UTR were on average smaller, while those of the OCR were even larger. When we repeated the heritability partitioning with TC approaches without LDMS stratification, we obtained higher contributions for all functional categories (Table 2; Additional file 1: Tables S24-25). For example, when using GREML, the following increases were observed: + 14% for CDS-UTR, + 45% for IOR, + 29% for UDR, + 5% for IGR and + 28% for OCR. These values are 1.5 times higher or more for all categories. The sum of the contributions estimated with TC approaches corresponded to more than 200% of the total genetic variance (Table 2). Similar results were obtained using a TC-GREML with LDMS stratification but convergence was not systematically achieved with the GREML approach.

**Table 2:** Average %SNP heritability and heritability enrichment estimated for five functional groups and for six traits measured in Belgian Blue beef cattle. Values were estimated by fitting all components simultaneously with multiple classes (MC) or each component in turn with two component (TC) models and without correction for LDMS, and using BayesRR-RC or GREML (values in the parentheses correspond to the GREML partitioning when the GRMs were computed using the first rules from VanRaden [29])

Annotation	%SNP heritability (MC)		Heritability enrichment (MC)		%SNP heritability (TC)		Heritability enrichment (TC)	
	GREML	BayesRR-RC	GREML	BayesRR-RC	GREML	BayesRR-RC	GREML	BayesRR-RC
CDS-UTR	18.8 (14.0)	16.1	30.9 (23.0)	26.4	33.0	33.8	54.3	55.6
IOR	16.9 (16.0)	17.4	0.6 (0.5)	0.6	62.2	58.8	2.1	1.9
UDR	0.0 (1.2)	8.5	0.0 (0.2)	1.6	28.7	28.1	5.4	5.3
IGR	10.4 (9.0)	12.7	0.2 (0.2)	0.2	15.5	18.4	0.3	0.3
OCR	53.9 (59.8)	45.3	5.1 (5.6)	4.3	82.1	70.9	7.7	6.7
Sum	100.0	100.0			221.5	210.0		

*CDS* Coding sequence, *IGR* Intergenic regions, *IOR* Intronic regions, *OCR* Open chromatin regions, *UDR* Up-stream and down-stream regions, *UTR* 3' and 5' UTR

CDS and UTR classes were merged

## Discussion

### Limitations of heritability partitioning approaches in livestock species

We herein evaluated the accuracy of GREML and BayesRR-RC in partitioning heritability according to functional classes, defined mainly on the basis of their position relative to genes and transcripts. Importantly, we evaluated the methods in a typical livestock population with reduced effective population size, high levels of relatedness and inbreeding, under intensive selection, and with high levels of long-range LD. The GREML approach has already been used in such livestock populations, for example in cattle [10, 11, 32–34]. Most often, this partitioning method was applied without an evaluation of its bias and accuracy in such context. However, differences in population structure and their impact on genome structure (e.g. LD patterns) could affect the precision and accuracy of the methods. For example, in humans, the methods have been evaluated by carefully filtering out pairs of individuals with levels of relatedness greater than 0.025 [12]. In livestock, a large fraction of pairs of individuals would have levels above such a threshold. Recently, Cai et al. [35] conducted a study to evaluate different GREML approaches for estimating heritability enrichment in a cattle population. They used data from 2,000 Holstein bulls imputed for about 700,000 markers, and mainly evaluated the accuracy of the estimators for three different MAF categories. Although some models gave unbiased results, biased estimators were observed when parameters from the simulated and fitted models did not match [35]. In particular, they found that estimated enrichment values were biased when CVs were enriched in rare alleles and that using LD scores calculated in too large windows resulted in biased estimates. We herein performed a simulation approach based on a large cohort of individuals. Importantly, our data were imputed at the whole-genome sequence level, providing a finer resolution for annotation. Compared to the study by Cai et al. [35], we included more functional annotation groups, including information from a recently published ATAC-SEQ peak catalogue [5], and we explored more scenarios (CVs could be enriched as a function of MAF, LD score and functional annotation). Using this approach, we first observed that in relatively simple scenarios (no stratification by MAF or LD, with CVs enriched for a single functional category), the methods were unbiased, but that the estimates showed high levels of variation. Note that when simulations were performed using the whole genome, even higher levels of variation were observed with the GREML approach (data not shown). When CVs were enriched in a particular MAF or LD score category, it was necessary to stratify the GREML or BayesRR-RC accordingly to obtain unbiased results (i.e., using a LDMS approach), consistent with findings in humans [12]. When GRMs were not defined for different MAF or LD groups, biased partitioning was indeed obtained. Importantly, the LD or MS groups fitted in the partitioning methods should match those that are truly enriched in CVs, an information that is rarely known. Other elements could further bias the results, such as the relationship between the MAF or LD scores of CVs and the magnitude of their effects, as previously highlighted by Speed et al. [31] or Cai et al. [35]. For example, the fitted GRMs could assume equal SNP contribution to the genetic variance (rare alleles having then larger effects) or comparable effect sizes for all SNPs regardless of MAF (common SNPs having higher contribution to the genetic variance), whereas the true relationship between CVs and MAF could be different. Simulation results indicated that such differences between simulated and fitted architecture can sometimes be compensated by the use of an LDMS approach. Next, we ran simulations in which multiple functional categories contributed to the phenotypic variation with different levels of enrichment. The estimators still showed high levels of variation, especially for the classes with few variants, but we also observed systematic biases due to confounding between some functional categories, the strongest between OCR and UDR. Estimators were better for variants in IGR, as their GRM was less similar to the GRM of other categories. This is important because it implies that confounding is higher for functional categories that are expected to contribute most to the genetic variance, and thus their estimates are less precise. We also tested a strategy estimating the %SNP heritability of each category individually (running one TC-GREML per category) and observed very strong biases, probably due to confounding. The estimated %SNP heritabilities were greatly overestimated for most categories. Although this two-component strategy reduces computational costs and allows fitting a LDMS model, it is therefore not recommended. This is an important observation as this is a common strategy [10, 11, 32, 33, 35–37].

The high levels of variation in heritability enrichment estimates could also be due to the similarity between GRMs from different functional or LDMS categories, or due to LD between neighboring SNPs from different categories. This problem is likely to be more severe in livestock species because the additive genetic relationships *r*_*xy*_ between pairs of individuals *x* and *y* are spread over a wider range, including unrelated individuals (*r*_*xy*_ = 0), half-sibs (*r*_*xy*_ = 0.25), full-sibs (*r*_*xy*_ = 0.5), parent-offspring (*r*_*xy*_ = 0.5) and even monozygotic twin (*r*_*xy*_ = 1) pairs. The high levels of relatedness will drive the correlations between elements from the different GRMs and may mask more subtle correlations due to short-range LD between SNPs. It has been shown that the properties of heritability estimators are different when individuals are unrelated and LD is high only at short distances [38]. When GRMs from different fitted categories are more distant, the problem of bias due to confounding between categories is likely to be less. This would be the case, for example, in studies evaluating the contribution from each autosome separately [34, 39, 40], or from specific chromosomes of interest such as the sex chromosomes [41]. For example, GRM from sex chromosomes are based on different segregation rules and are less correlated with GRMs obtained from autosomes [39, 42]. Similarly, relationship matrices could be estimated for mitochondria or chloroplasts in plants to assess their contribution to the genetic variance.

### Comparison of evaluated methods

Our evaluation focused on two methods, GREML and BayesRR-RC. For GREML, we estimated the GRM using the rules from Yang et al. [25]. Different GRM construction rules can lead to different estimators. For example, the GRM may be based on different relationships between the variance of marker effects and their MAF (by defining a parameter called “α”), LD score, or their genotyping accuracy [31, 43]. The accuracy of GREML with different values of α has previously been evaluated in a livestock population by Cai et al. [35]. In preliminary tests, we obtained less accurate estimates with the LDAK-Thin model recommended for non-human organisms [43], and therefore selected original rules from Yang et al. [25], assuming that each marker has an equal expected contribution to heritability (i.e., independent of MAF), to construct our GRM. Nevertheless, in additional simulations, we observed that a mismatch between the function used to compute the GRM and the simulated relationship between CV effects and their MAF or LD scores could bias the results, more so when the relationship with LD scores was suboptimal and with GREML (data not shown). Unfortunately, the relationships remain unknown and, based on our results, the use of BayesRR-RC and LDMS models is recommended in such situations. The LD score regression (LDSR) is another method that allows heritability partitioning [9]. It is computationally efficient because it relies on summary statistics. Nevertheless, heritability estimates from LDSR have higher standard errors than those from GREML [31, 44], and the approach has not been shown to be more efficient than GREML or BayesRR-RC in several studies [13, 45]. The properties of LDSR need to be evaluated in livestock populations, where the extent of LD is very different from humans and where markers may be in linkage equilibrium with CVs due to the presence of high levels of relatedness. For instance, Xiang et al. [37] obtained poor results with LDSR in dairy cattle. In addition, obtaining summary statistics in livestock populations is more computationally demanding because LMM must be used for GWAS to correct for stratification and polygenic background. Due to these high computational requirements and based on previous comparison results, we did not evaluate LDSR in our study. Compared to BayesRR-RC, GREML produced more accurate results in the first set of simulations where OCR variants accounted for 50% of the heritability. In similar cases, LDMS models are recommended to obtain unbiased results. However, with many different fitted components, 500 iterations of the EM algorithm were sometimes insufficient to achieve convergence. These problems could be reduced by fitting a two-component model, but this produced biased results (see above). When we fitted models with multiple functional categories, BayesRR-RC outperformed the GREML approach. However, Bayes-RR-RC has higher computational costs and the number of iterations that can be run is relatively small. Convergence diagnostic plots and comparisons with longer chains suggest that this number of iterations already provides good estimates for most parameters although these had high levels of variation (see Additional file 2: Figures S10 and S11). This is consistent with the results of Orliac et al. [28] who concluded that less than 5,000 iterations are required to estimate variance components and for genomic predictions. In the most complex scenarios, the estimator for some parameters was not fully stabilized after 5,000 iterations (Additional file 2: Figure S11). This suggests that more iterations may be required for livestock species due to the higher LD and relatedness levels. Nevertheless, comparisons of the results obtained with 5,000 versus 50,000 iterations for 25 simulations from 2 scenarios show that the distributions of the estimated parameters are very similar. Overall, we observed that, with a total of 5,000 iterations, Bayes-RR-RC performed better than GREML, but we cannot exclude that longer chains could further improve the results.

### Heritability partitioning for muscularity and height in Belgian Blue beef cattle

Despite the high standard errors in the simulations, the estimated heritability enrichments and their ranking remain informative, especially when averaged over multiple traits, as done in other studies [8, 9]. With both GREML and BayesRR-RC, variants present in OCR had by far the largest contribution to heritability (> 45%). Regulatory regions have also been shown to have the largest contribution to genetic variance for complex traits in humans [8] and to be important in cattle [10, 11]. Recently, Xiang et al. [37] evaluated that regulatory variants explained on up to 70% of the genetic variance in cattle. In terms of heritability enrichment, variants in coding regions had the highest average per-variant contribution to the heritability (> 25-fold on average), variants in the OCR also showed substantial enrichment (~ fivefold), whereas intronic and intergenic variants had enrichment values below 1 (0.6 and 0.2-fold, respectively). This ranking is in line with expectations and is consistent with results obtained in several studies of complex traits in humans [8, 9]. The observation of large effects associated with variants in coding regions is in agreement with the findings of Gualdrón Duarte et al. [23], who identified several coding variants associated with the same traits and accounting for a large proportion of the genetic variance. Heritability partitioning could be refined by using more specific functional classes such as coding variants or eQTLs, but care must be taken as we have shown the limitations of partitioning approaches when too small or too many categories were fitted. Similarly, heritability enrichment could be applied to other types of categories such as conservation scores, differentiation scores, evidence of selection, or age of alleles.

## Conclusions

Here we have shown that heritability partitioning approaches should be used cautiously in livestock populations and that accuracy assessment is strongly recommended. Estimators were particularly imprecise for small categories, so models with too many and small functional categories should not be used. In addition, two-component approaches that fit only one functional category at a time produced biased estimates and should not be used. Nevertheless, the estimates and their ranking were still informative about the contribution of the functional classes we fitted. We therefore applied the methods to real phenotypes for muscular development and height. We estimated that, on average, variants in open chromatin regions had a higher contribution to the genetic variance, while variants in coding regions had the strongest individual effects. Conversely, variants in intergenic or intronic regions showed lower levels of enrichment. The results are consistent with those obtained in humans.

### Supplementary Information

Additional file 1. Supplementary Tables. Table S1. %SNP heritability estimation when variants in open chromatin regions account for 50% of the heritability. Table S2. %SNP heritability estimation when causal variants are enriched in low MAF variants. Table S3. %SNP heritability estimation when causal variants are enriched in low LD score variants. Table S4. %SNP heritability estimation when causal variants are enriched in low MAF and LD score variants. Table S5. %SNP heritability estimation when causal variants are enriched in common variants. Table S6. %SNP heritability estimation when causal variants are enriched in high LD score variants. Table S7. %SNP heritability estimation when causal variants are enriched in low MAF and high LD score variants. Table S8. %SNP heritability estimation with multiple functional categories when the variants in coding sequence account for 100% of the heritability. Table S9. %SNP heritability estimation with multiple functional categories when the variants in intronic regions account for 100% of the heritability. Table S10. %SNP heritability estimation with multiple functional categories when the variants in upstream and downstream regions account for 100% of the heritability. Table S11. %SNP heritability estimation with multiple functional categories when the variants in intergenic regions account for 100% of the heritability. Table S12. %SNP heritability estimation with multiple functional categories when the variants in open chromatin regions account for 100% of the heritability. Table S13. %SNP heritability estimation with multiple functional categories when the variants from different functional classes had equal contribution. Table S14. %SNP heritability estimation in complex simulation scenarios where SNPs from different functional classes had variable contributions (scenario I). Table S15. %SNP heritability estimation in complex simulation scenarios where SNPs from different functional classes had variable contributions (scenario II). Table S16. %SNP heritability estimation in complex simulation scenarios where SNPs from different functional classes had variable contributions (scenario III). Table S17. Mean Absolute Error of %SNP heritability estimates across the three complex simulation scenarios where SNPs from different functional classes had variable contributions. Table S18. %SNP heritability estimation with a two-component approach when the variants from different functional classes had equal contribution. Table S19. %SNP heritability estimation with a two-component approach in complex simulation scenarios where SNPs from different functional classes had variable contributions (scenario I). Table S20. %SNP heritability estimation with a two-component approach in complex simulation scenarios where SNPs from different functional classes had variable contributions (scenario II). Table S21. %SNP heritability estimation with a two-component approach in complex simulation scenarios where SNPs from different functional classes had variable contributions (scenario III). Table S22. Mean Absolute Error of %SNP heritability estimates using a two-component approach across the three complex simulation scenarios where SNPs from different functional classes had variable contributions. Table S23. Correlations between genomic relationship matrices from different functional categories. Table S24. %SNP heritability estimation for the measured phenotypes. Table S25. Heritability enrichment estimation for the measured phenotypes. Table S26. %SNP heritability and heritability enrichment estimation using a LDMS two component approach for the measured phenotypes.

Additional file 2. Supplementary Figures Figure S1. Estimation of %SNP heritability when causal variants are enriched in specific MAF or LD score categories. Variants in open chromatin regions (OCR) accounted for 50% of heritability. Causal variants were enriched in A) common variants (MAF > 0.20), B) high LD variants (LD score above the 3rd quartile), and C) low MAF (MAF < 0.05) and high LD (LD score above the 3rd quartile) variants. The %SNP heritability was estimated with GREML and BayesRR-RC. The methods were applied without correction for MAF or LD score (noLDMS), and with MAF stratified (MS), LD stratified (LDS) and both MAF and LD stratified (LDMS) approaches. Figure S2. Estimation of %SNP heritability using different GRM computation methods and for the two scenarios where SNP effect size is a function of allele frequency. Simulation rule 1: SNP effects increase as allele frequencies decrease (corresponding to the default rule). Simulation rule 2: SNP effects are drawn from the same distribution regardless of allele frequency (corresponding to the rules proposed by VanRaden [29]). Partitioning GRM rule 1: GRMs used in the heritability partitioning are computed using the default rules from GCTA. Partitioning GRM rule 2: GRMs used in heritability partitioning are computed using the VanRaden rules from. Figure S3. Estimation of %SNP heritability when causal variants are enriched in a single functional annotation class. Causal variants were located in A) upstream and downstream regions (UDR), B) intergenic regions (IGR), and C) intronic regions (IOR). The %SNP heritability was estimated using GREML and BayesRR-RC with the following functional classes: coding sequence (CDS), 3’ and 5’ UTRs (UTR), UDR, IOR, IGR and open chromatin regions (OCR). Figure S4. Scatterplot of estimated versus true heritability enrichment across simulation scenarios where SNPs from different functional classes contribute to genetic variance. The comparison is made separately for each functional class. Heritability enrichment was estimated using GREML and BayesRR-RC with the following functional classes: coding sequence (CDS), 3’ and 5’ UTRs (UTR), upstream and downstream regions (UDR), intronic regions (IOR), intergenic regions (IGR) and open chromatin regions (OCR). Figure S5. Estimation of %SNP heritability of variants in intergenic regions (IGR) using a two-component strategy. Estimation was performed in complex simulation scenarios in which SNPs from multiple functional classes contribute to genetic variance (Panel A for the scenario without enrichment and Panels B-D for complex scenarios 1 to 3, respectively). Heritability enrichment was estimated using GREML and BayesRR-RC with the following two functional classes (IGR versus other categories). In addition, methods were run with unstratified (US), MAF stratified (MS), LD stratified (LDS) and both MAF and LD stratified (LDMS) approaches. Figure S6. Estimation of %SNP heritability of variants in coding sequence (CDS) using a two-component strategy. Estimation was performed in complex simulation scenarios in which SNPs from multiple functional classes contribute to genetic variance (Panel A for the scenario without enrichment and Panels B-D for complex scenarios 1 to 3, respectively). Heritability enrichment was estimated using GREML and BayesRR-RC with the following two functional classes (CDS versus other categories). In addition, methods were run without correction for MAF or LD score (noLDMS), and with MAF stratified (MS), LD stratified (LDS) and both MAF and LD stratified (LDMS) approaches. Figure S7. Estimation of %SNP heritability of variants in intronic regions (IOR) using a two-component strategy. Estimation was performed in complex simulation scenarios in which SNPs from multiple functional classes contribute to genetic variance (Panel A for the scenario without enrichment and Panels B-D for complex scenarios 1 to 3, respectively). Heritability enrichment was estimated using GREML and BayesRR-RC with the following two functional classes (IOR versus other categories). In addition, methods were run without correction for MAF or LD score (noLDMS), and with MAF stratified (MS), LD stratified (LDS) and both MAF and LD stratified (LDMS) approaches. Figure S8. Estimation of %SNP heritability of variants in upstream and downstream regions (UDR) using a two-component strategy. Estimation was performed in complex simulation scenarios in which SNPs from multiple functional classes contribute to genetic variance (Panel A for the scenario without enrichment and Panels B-D for complex scenarios 1 to 3, respectively). Heritability enrichment was estimated using GREML and BayesRR-RC with the following two functional classes (UDR versus other categories). In addition, methods were run without correction for MAF or LD score (noLDMS), and with MAF stratified (MS), LD stratified (LDS) and both MAF and LD stratified (LDMS) approaches. Figure S9. Scatterplot of estimated versus true %SNP heritability when using a two-component strategy. Estimates were compared across simulation scenarios where SNPs from different functional classes contribute to genetic variance. The contribution for each category is shown in Table 1. The comparison is made separately for each functional class. %SNP heritability was estimated using GREML and BayesRR-RC with the following two functional classes (one versus other categories) and a MAF and LD stratified (LDMS) approach. Fitted functional categories were coding sequence (CDS), 3’ and 5’ UTRs (UTR), upstream and downstream regions (UDR), intronic regions (IOR), intergenic regions (IGR) and open chromatin regions (OCR). Figure S10. Comparison of BayesRR-RC results obtained with 5,000 versus 50,000 iterations in a simple scenario. The model was run on data from a simple scenario where OCR contributed to 50% of the genetic variance. The 5,000 iterations correspond to the values used in the present study (burn-in from iterations 1–2,000), while 50,000 iterations correspond to a longer run (burn-in from iterations 1–5,000). A) Estimated %SNP heritability per iteration. Iterations used for parameter estimation in the standard run are delimited by the two blue dashed lines located at iterations 2,001 and 5,000. B) Distribution of %SNP heritability estimates in iterations 2,001–5,000 (standard run) and 5,001–50,000 (long run). C) %SNP heritability estimates for 25 simulations estimated using BayesRR-RC with 5,000 versus 50,000 iterations. Figure S11. Comparison of BayesRR-RC results obtained with 5,000 versus 50,000 iterations in the first complex scenario. The 5,000 iterations correspond to the values used in the present study (burn-in from iterations 1–2,000), while 50,000 iterations correspond to a longer run (burn-in from iterations 1–5,000). A) Estimated %SNP heritability per iteration for the six components. Iterations used for parameter estimation in the standard run are delimited by the two blue dashed lines located at iterations 2,001 and 5,000. B) %SNP heritability estimates for the six components estimated using BayesRR-RC with 5,000 versus 50,000 iterations in 25 simulations.

## Data Availability

Raw genotypes and phenotypes data are part of a reference population used for genomic selection and have commercial value. Therefore, restrictions apply to their availability and they are not publicly available. The authors can be contacted for a reasonable request.

## Abbreviations

AI: Average Information
ATAC-seq: The assay for transposase-accessible chromatin with sequencing
CDS: Coding sequence
CVs: Causal variants
EM: Expectation Maximization
GREML: Genomic restricted maximum likelihood
GRM: Genomic relationship matrix
GTR: General Transfer Format
IGR: Intergenic
indels: Insertion and deletions
IOR: Intronic
LD: Linkage disequilibrium
LDMS: LD and MAF-stratified
LDRS: LD regression score
LDS: LD-stratified
MAF: Minor allele frequency
MC: Multiple component
MS: MAF-stratified
noLDMS: Without correction for MAF or LD score
RSME: Residual mean square error
TC: Two component
TSS: Transcription start sites
TTS: Transcription termination sites
UDR: Upstream or downstream of genes
US: Unstratified
UTR: Untranslated regions
