# Supplementary material for: Evaluation of heritability partitioning approaches in livestock populations
Source: BMC Genomics. 2024 Jul 13;25:690. doi: 10.1186/s12864-024-10600-y (PMC11246585; doi:10.1186/s12864-024-10600-y)
Supplement: Supplementary file 2 — Additional file 2. Supplementary Figures Figure S1. Estimation of %SNP heritability when causal variants are enriched in specific MAF or LD score categories. Variants in open chromatin regions (OCR) accounted for 50% of heritability. Causal variants were enriched in A) common variants (MAF > 0.20), B) high LD variants (LD score above the 3rd quartile), and C) low MAF (MAF < 0.05) and high LD (LD score above the 3rd quartile) variants. The %SNP heritability was estimated with GREML and BayesRR-RC. The methods were applied without correction for MAF or LD score (noLDMS), and with MAF stratified (MS), LD stratified (LDS) and both MAF and LD stratified (LDMS) approaches. Figure S2. Estimation of %SNP heritability using different GRM computation methods and for the two scenarios where SNP effect size is a function of allele frequency. Simulation rule 1: SNP effects increase as allele frequencies decrease (corresponding to the default rule). Simulation rule 2: SNP effects are drawn from the same distribution regardless of allele frequency (corresponding to the rules proposed by VanRaden [29]). Partitioning GRM rule 1: GRMs used in the heritability partitioning are computed using the default rules from GCTA. Partitioning GRM rule 2: GRMs used in heritability partitioning are computed using the VanRaden rules from. Figure S3. Estimation of %SNP heritability when causal variants are enriched in a single functional annotation class. Causal variants were located in A) upstream and downstream regions (UDR), B) intergenic regions (IGR), and C) intronic regions (IOR). The %SNP heritability was estimated using GREML and BayesRR-RC with the following functional classes: coding sequence (CDS), 3’ and 5’ UTRs (UTR), UDR, IOR, IGR and open chromatin regions (OCR). Figure S4. Scatterplot of estimated versus true heritability enrichment across simulation scenarios where SNPs from different functional classes contribute to genetic variance. The comparison is made separately for each func [file 12864_2024_10600_MOESM2_ESM.docx]

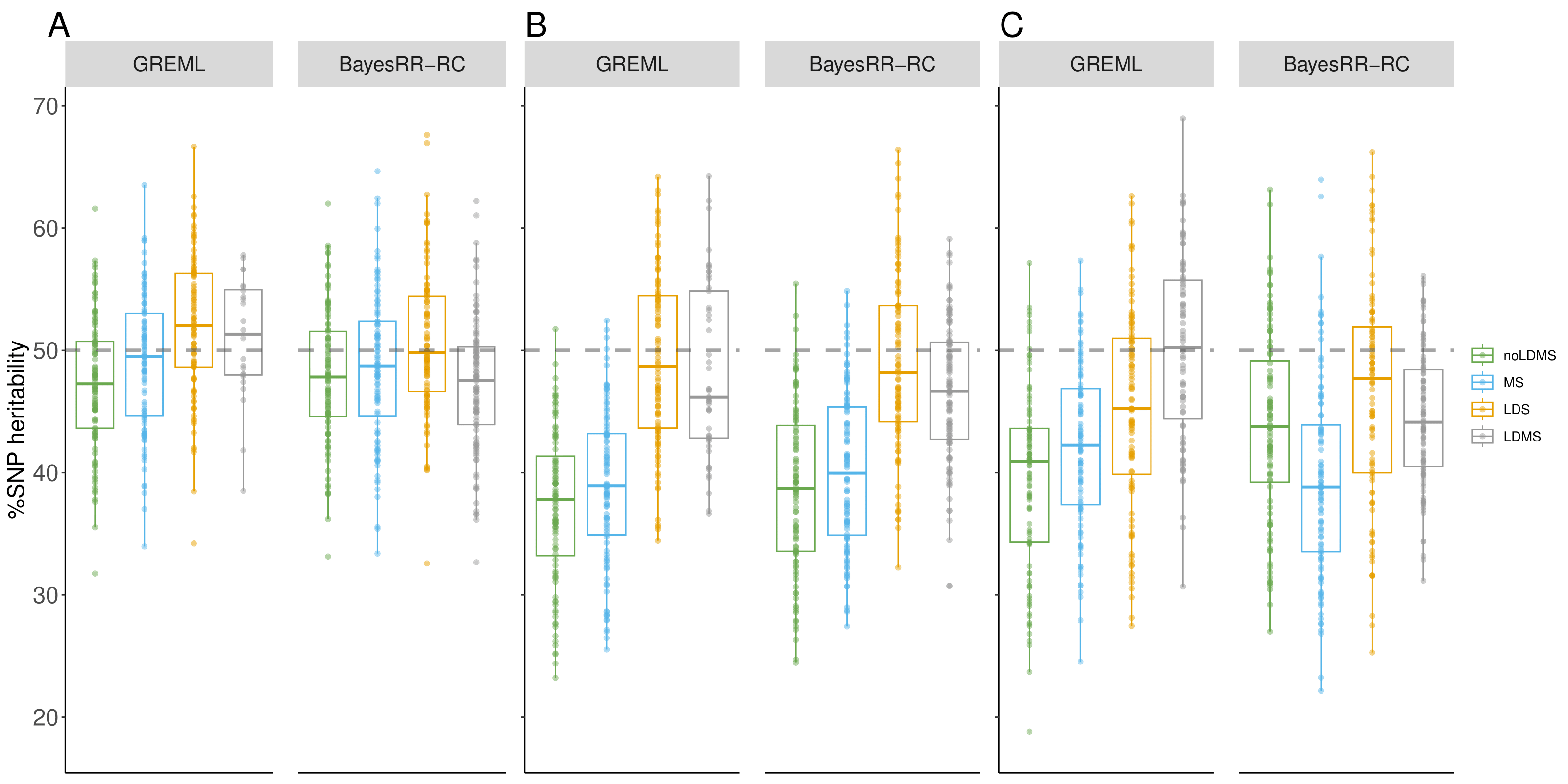


Figure S1. Estimation of %SNP heritability when causal variants are enriched in specific MAF or LD score categories. Variants in open chromatin regions (OCR) accounted for 50% of heritability. Causal variants were enriched in A) common variants (MAF > 0.20), B) high LD variants (LD score above the 3rd quartile), and C) low MAF (MAF <0.05) and high LD (LD score above the 3rd quartile) variants. The %SNP heritability was estimated with GREML for simulations using the whole genome (GREML – FULL) and with GREML and BayesRR-RC for simulations using a subset of the genome. The methods were applied without correction for MAF or LD score (noLDMS), and with MAF stratified (MS), LD stratified (LDS) and both MAF and LD stratified (LDMS) approaches.


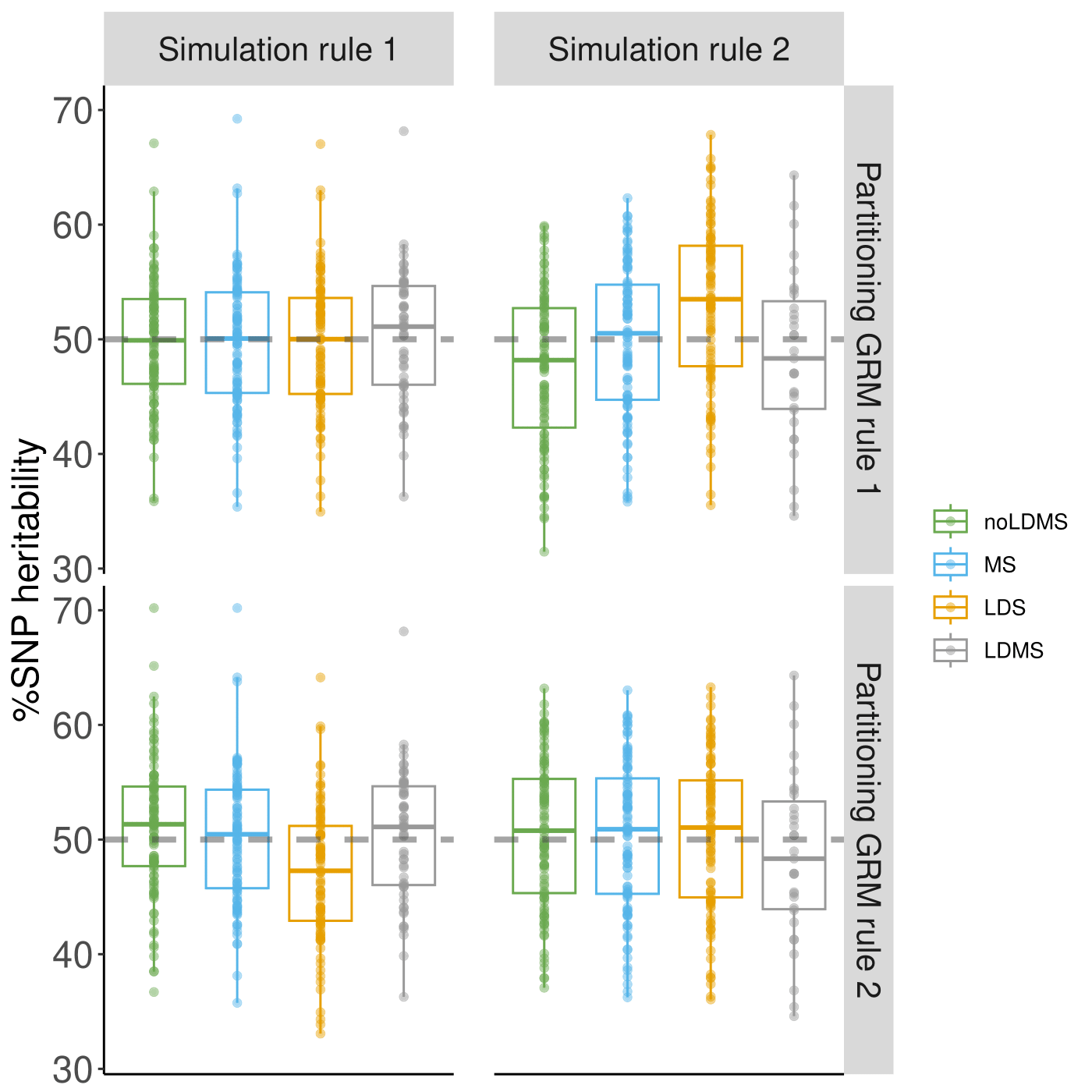


Figure S2. Estimation of %SNP heritability using different GRM computation methods and for the two scenarios where SNP effect size is a function of allele frequency. Simulation rule 1: SNP effects increase as allele frequencies decrease (corresponding to the default rule). Simulation rule 2: SNP effects are drawn from the same distribution regardless of allele frequency (corresponding to the rules proposed by VanRaden [29]). Partitioning GRM rule 1: GRMs used in the heritability partitioning are computed using the default rules from GCTA. Partitioning GRM rule 2: GRMs used in heritability partitioning are computed using the VanRaden rules from.


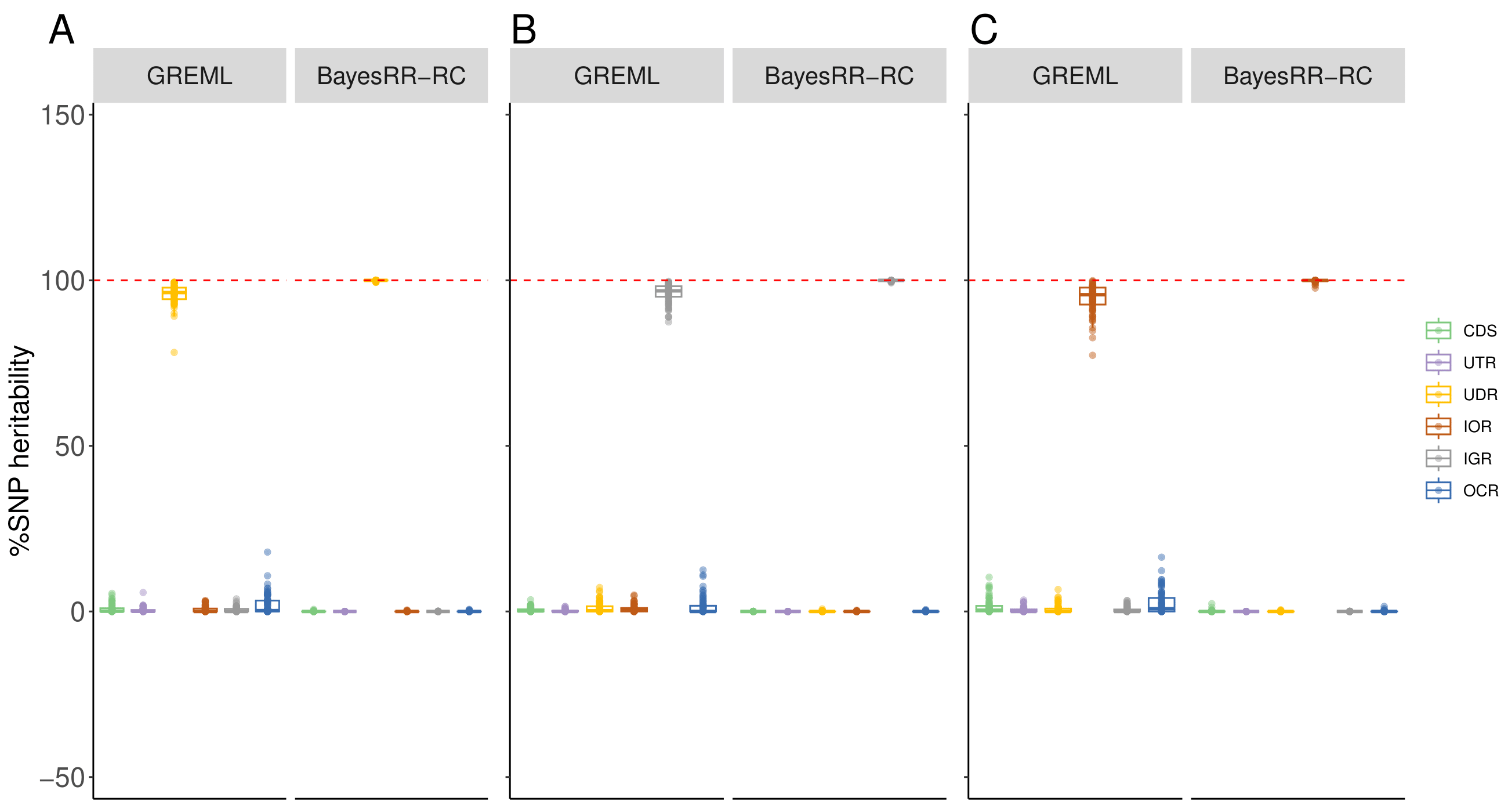


Figure S3. Estimation of %SNP heritability when causal variants are enriched in a single functional annotation class. Causal variants were located in A) upstream and downstream regions (UDR), B) intergenic regions (IGR), and C) intronic regions (IOR). The %SNP heritability was estimated using GREML and BayesRR-RC with the following functional classes: coding sequence (CDS), 3’ and 5’ UTRs (UTR), UDR, IOR, IGR and open chromatin regions (OCR).


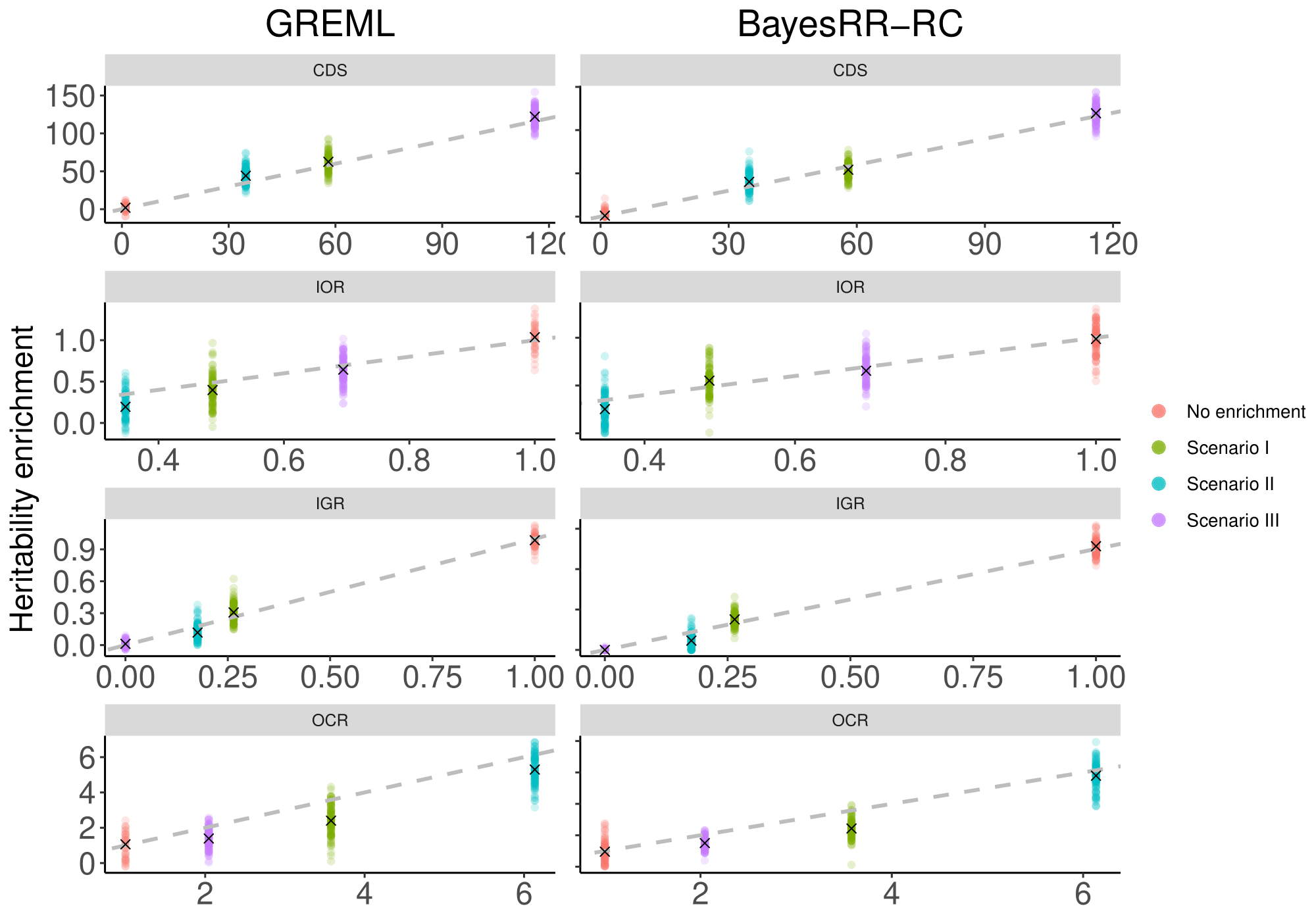


Figure S4. Scatterplot of estimated versus true heritability enrichment across simulation scenarios where SNPs from different functional classes contribute to genetic variance. The comparison is made separately for each functional class. Heritability enrichment was estimated using GREML and BayesRR-RC with the following functional classes: coding sequence (CDS), 3’ and 5’ UTRs (UTR), upstream and downstream regions (UDR), intronic regions (IOR), intergenic regions (IGR) and open chromatin regions (OCR).


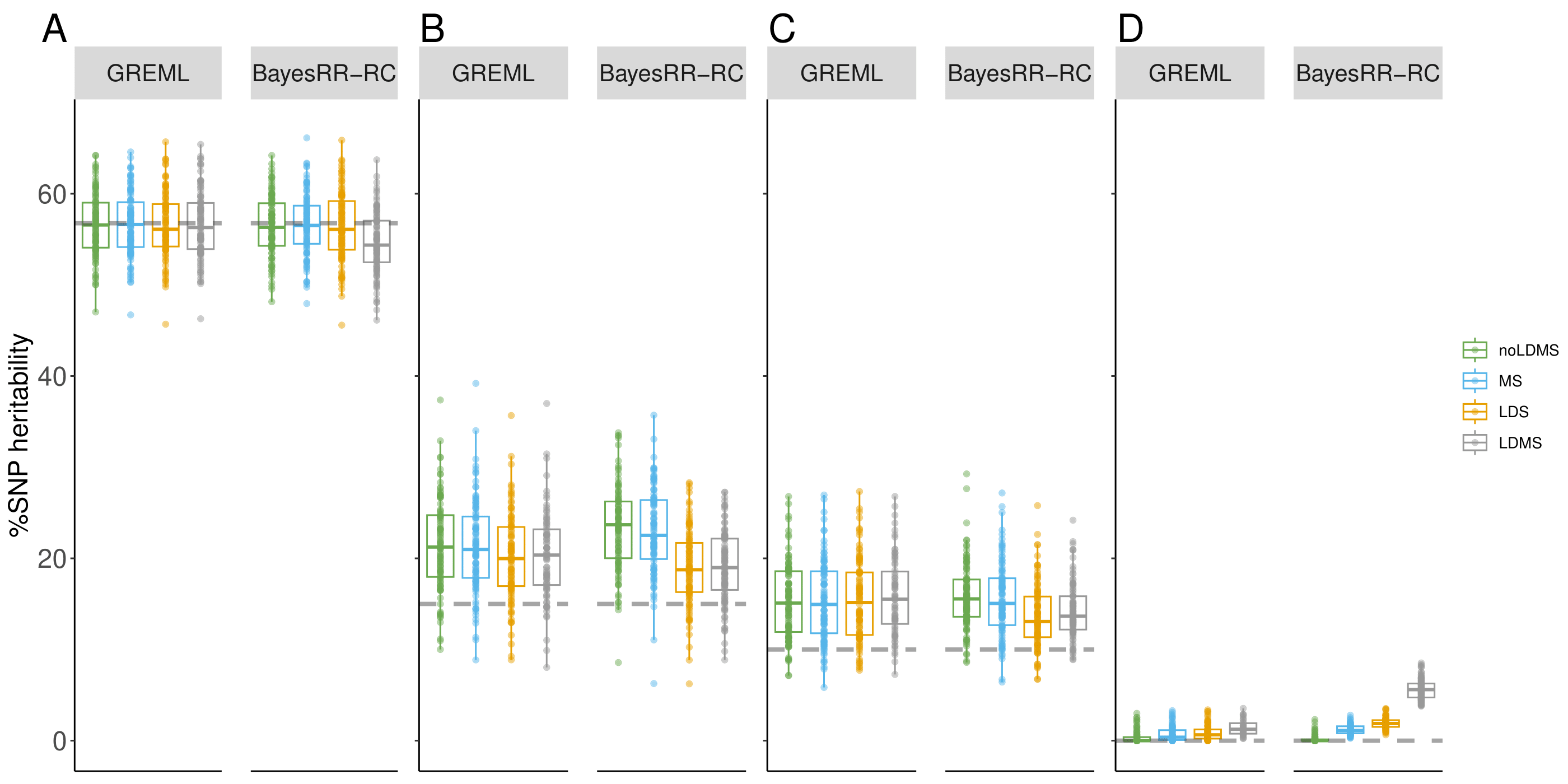


Figure S5. Estimation of %SNP heritability of variants in intergenic regions (IGR) using a two-component strategy. Estimation was performed in complex simulation scenarios in which SNPs from multiple functional classes contribute to genetic variance (Panel A for the scenario without enrichment and Panels B-D for complex scenarios 1 to 3, respectively). Heritability enrichment was estimated using GREML and BayesRR-RC with the following two functional classes (IGR versus other categories). In addition, methods were run with unstratified (US), MAF stratified (MS), LD stratified (LDS) and both MAF and LD stratified (LDMS) approaches.


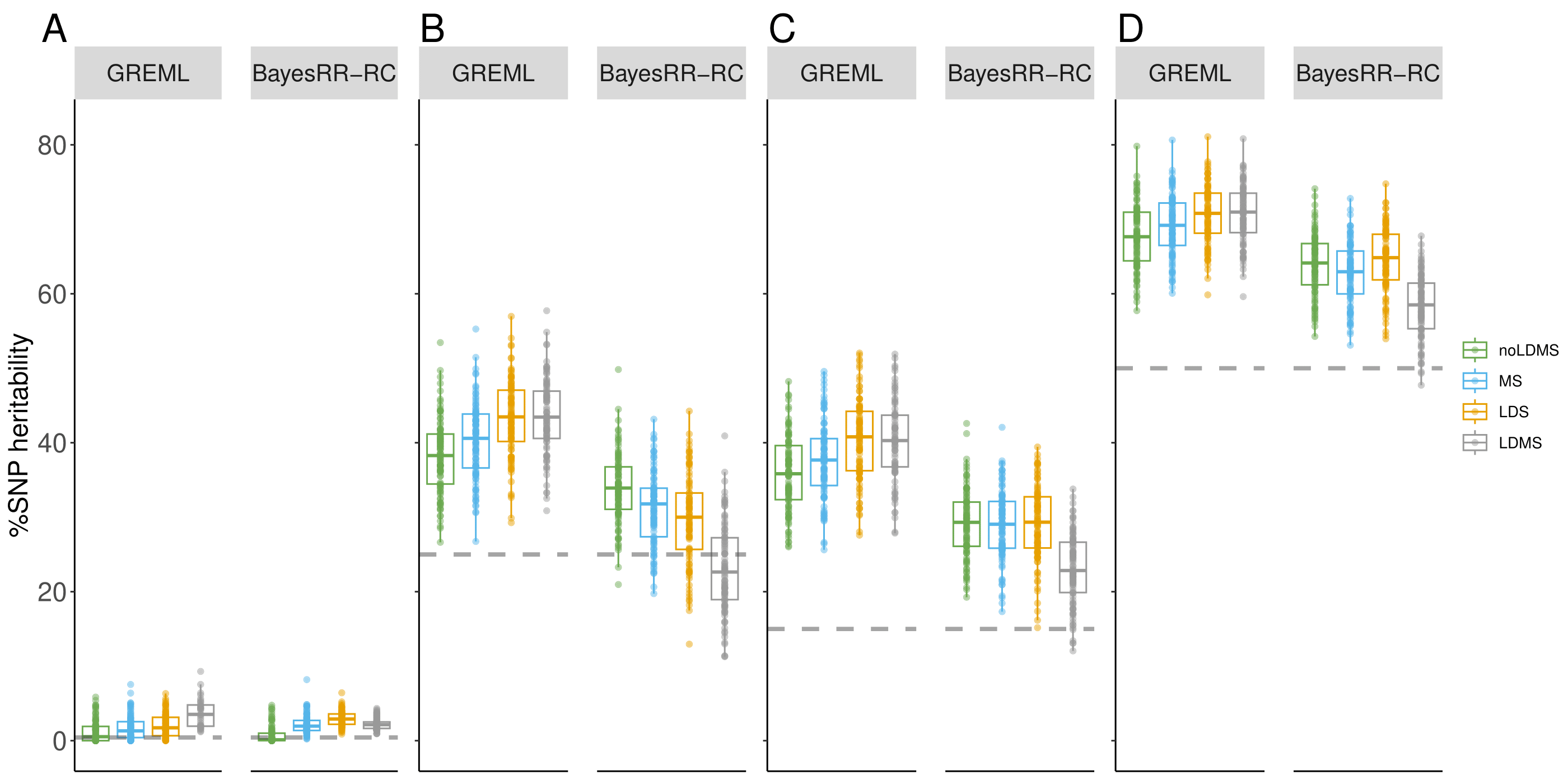


Figure S6. Estimation of %SNP heritability of variants in coding sequence (CDS) using a two-component strategy. Estimation was performed in complex simulation scenarios in which SNPs from multiple functional classes contribute to genetic variance (Panel A for the scenario without enrichment and Panels B-D for complex scenarios 1 to 3, respectively). Heritability enrichment was estimated using GREML and BayesRR-RC with the following two functional classes (CDS versus other categories). In addition, methods were run without correction for MAF or LD score (noLDMS), and with MAF stratified (MS), LD stratified (LDS) and both MAF and LD stratified (LDMS) approaches.


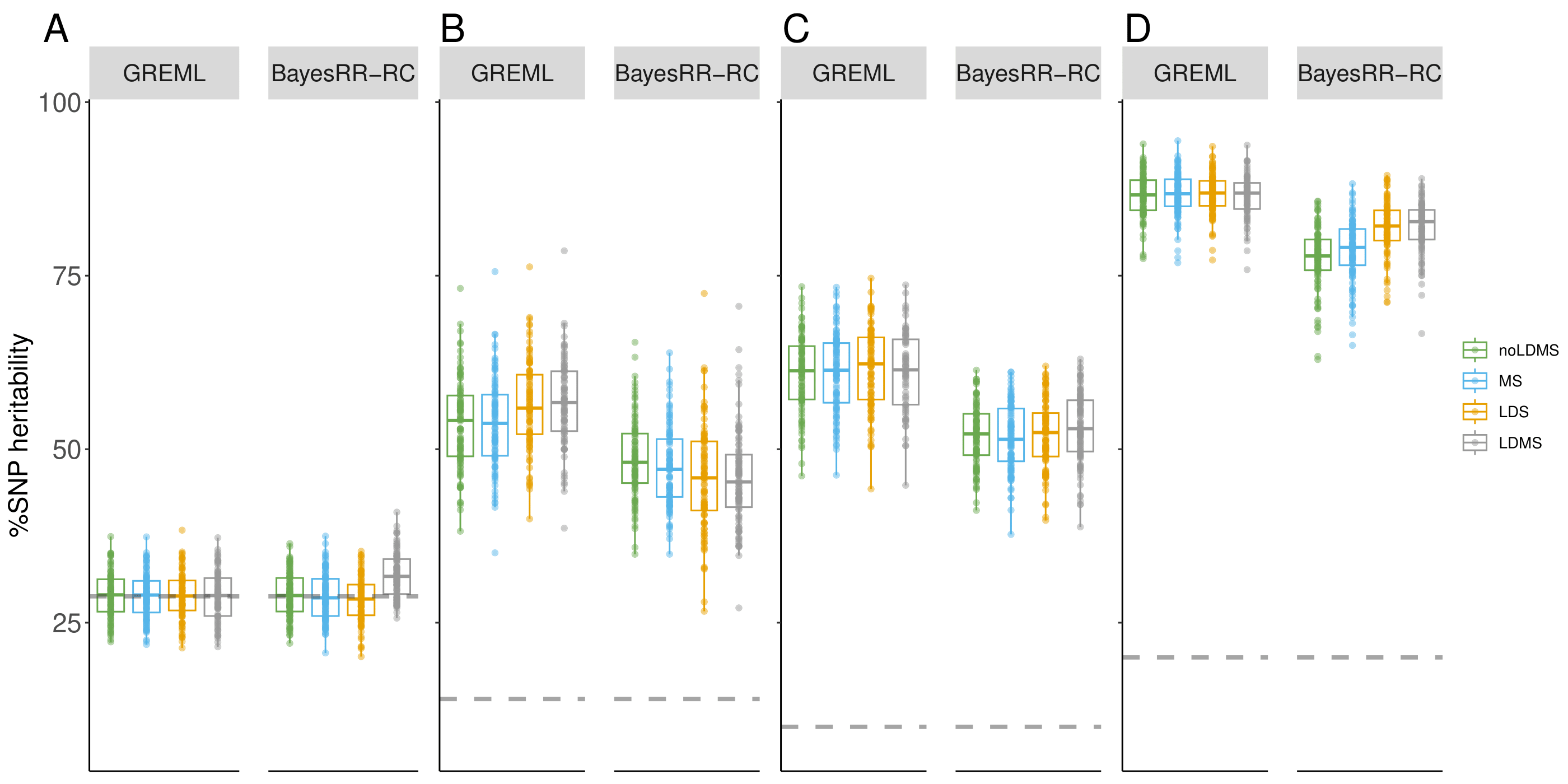


Figure S7. Estimation of %SNP heritability of variants in intronic regions (IOR) using a two-component strategy. Estimation was performed in complex simulation scenarios in which SNPs from multiple functional classes contribute to genetic variance (Panel A for the scenario without enrichment and Panels B-D for complex scenarios 1 to 3, respectively). Heritability enrichment was estimated using GREML and BayesRR-RC with the following two functional classes (IOR versus other categories). In addition, methods were run without correction for MAF or LD score (noLDMS), and with MAF stratified (MS), LD stratified (LDS) and both MAF and LD stratified (LDMS) approaches.


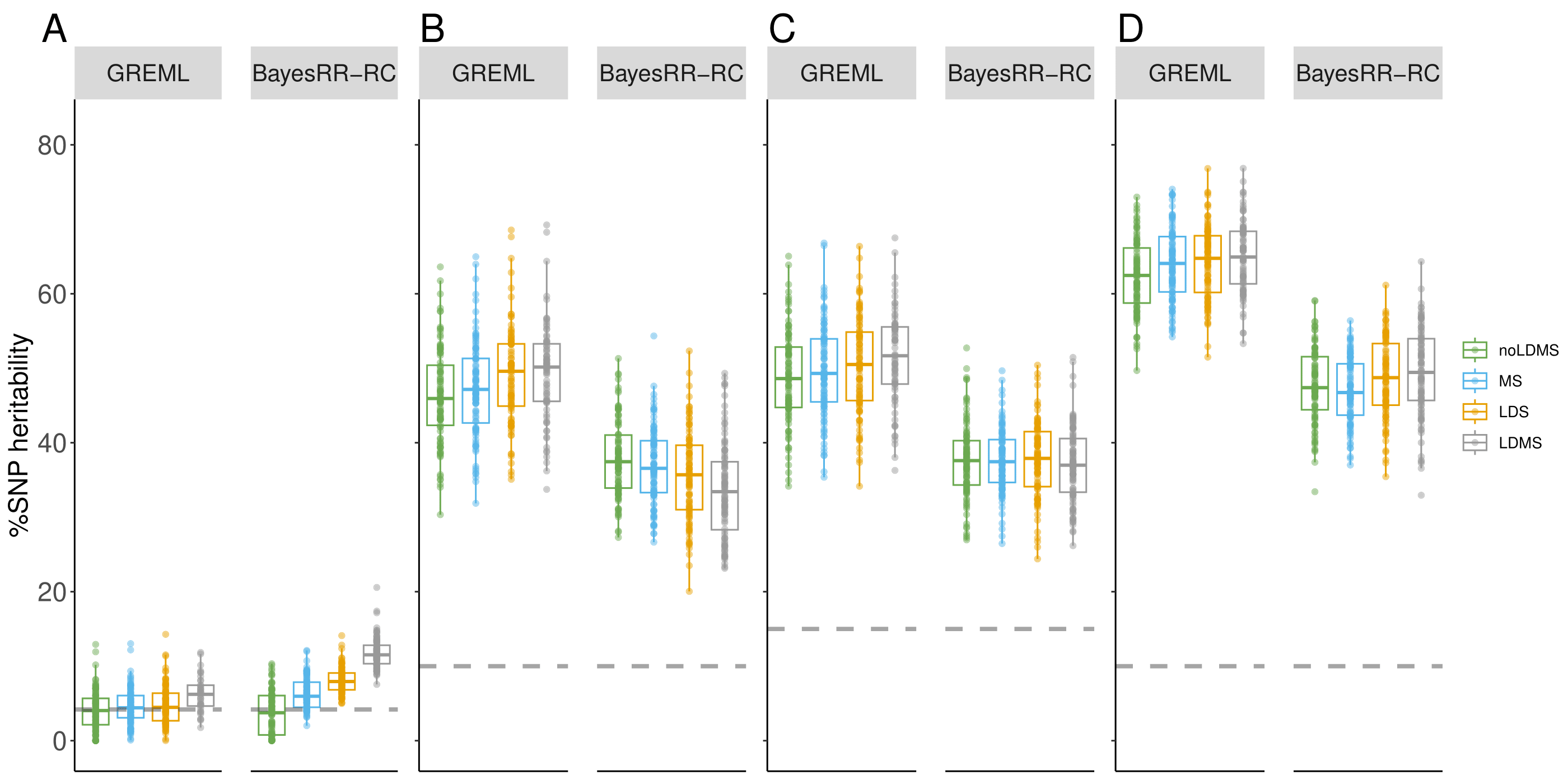


Figure S8. Estimation of %SNP heritability of variants in upstream and downstream regions (UDR) using a two-component strategy. Estimation was performed in complex simulation scenarios in which SNPs from multiple functional classes contribute to genetic variance (Panel A for the scenario without enrichment and Panels B-D for complex scenarios 1 to 3, respectively). Heritability enrichment was estimated using GREML and BayesRR-RC with the following two functional classes (UDR versus other categories). In addition, methods were run without correction for MAF or LD score (noLDMS), and with MAF stratified (MS), LD stratified (LDS) and both MAF and LD stratified (LDMS) approaches.


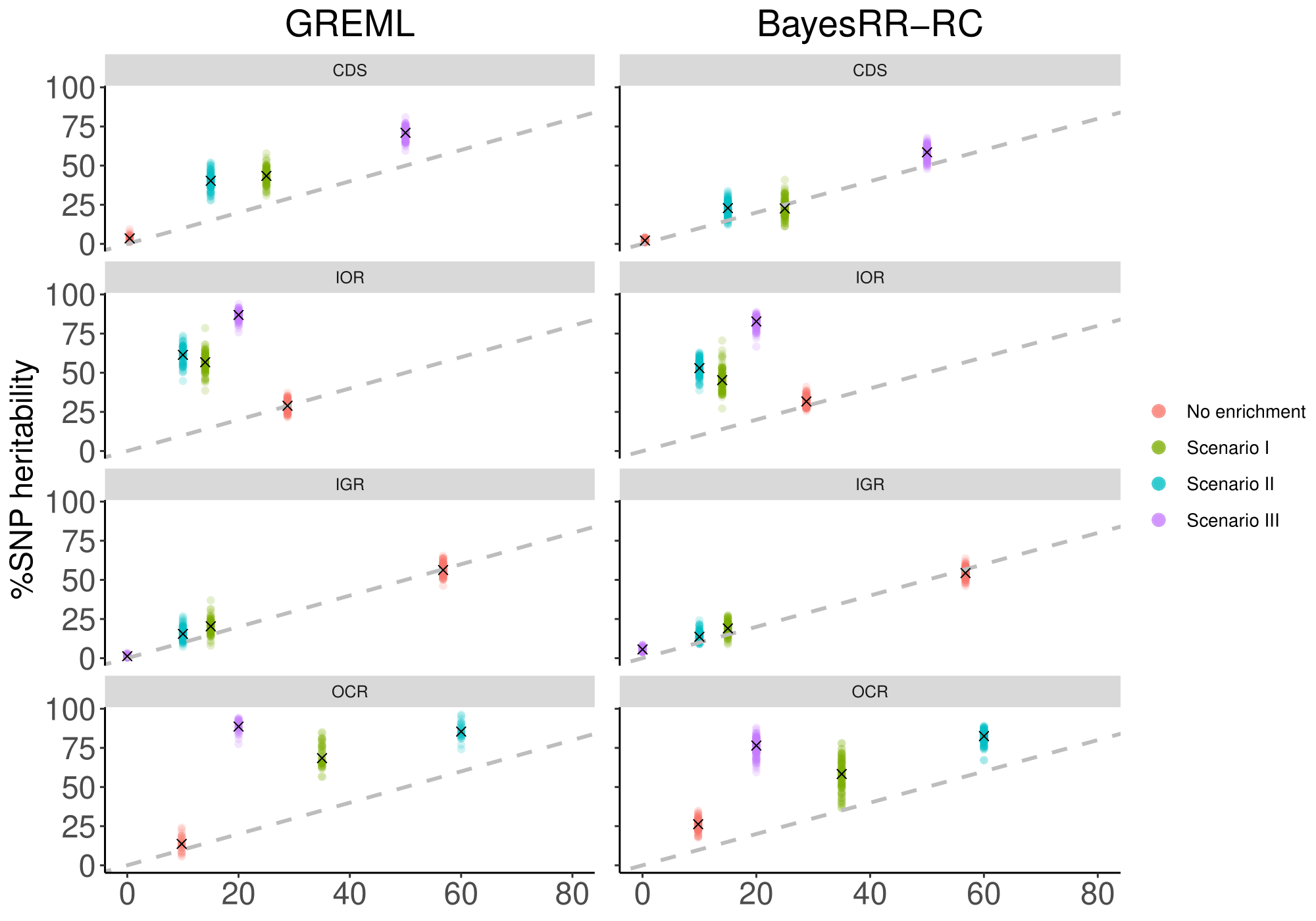


Figure S9. Scatterplot of estimated versus true %SNP heritability when using a two-component strategy. Estimates were compared across simulation scenarios where SNPs from different functional classes contribute to genetic variance. The contribution for each category is shown in Table 1. The comparison is made separately for each functional class. %SNP heritability was estimated using GREML and BayesRR-RC with the following two functional classes (one versus other categories) and a MAF and LD stratified (LDMS) approach. Fitted functional categories were coding sequence (CDS), 3’ and 5’ UTRs (UTR), upstream and downstream regions (UDR), intronic regions (IOR), intergenic regions (IGR) and open chromatin regions (OCR).


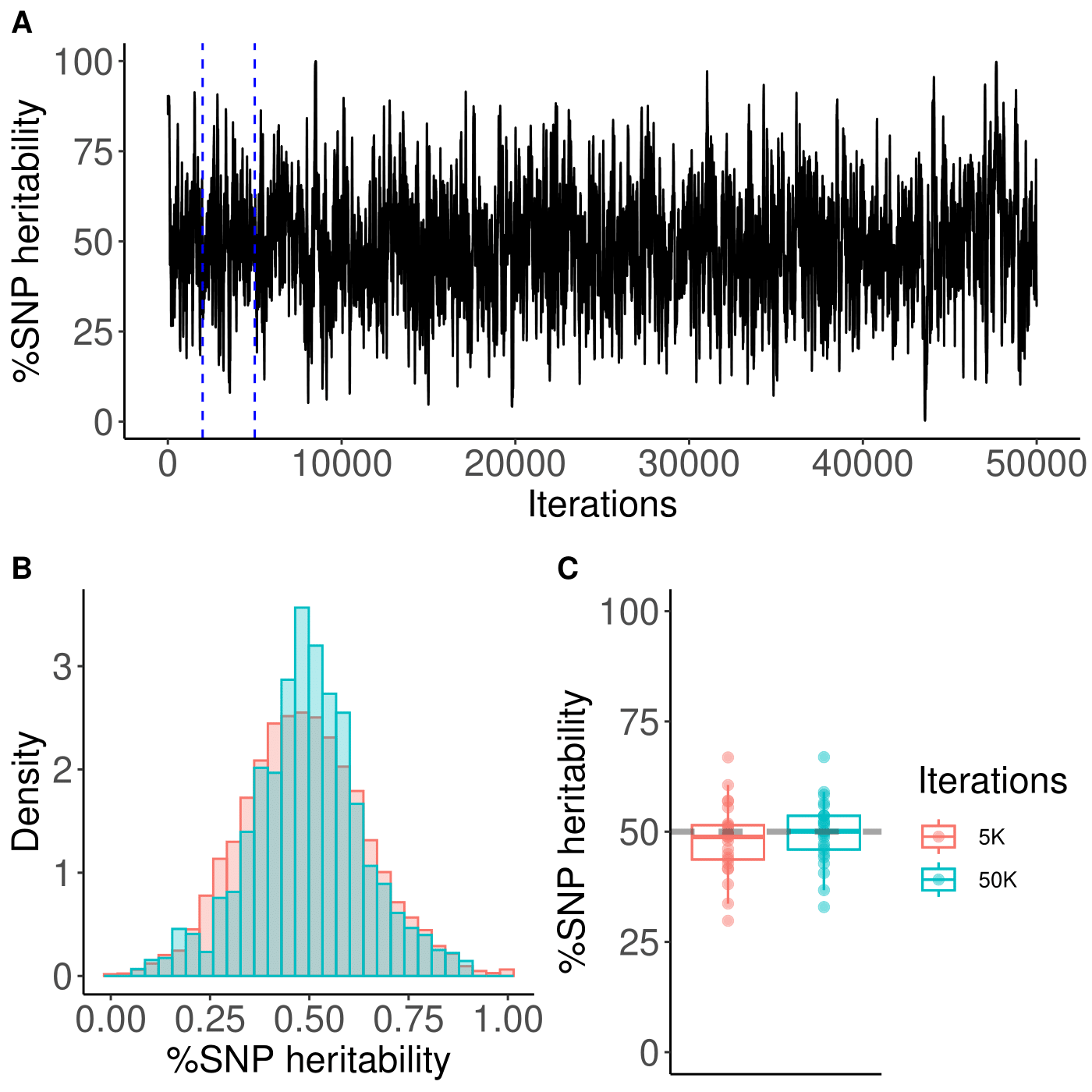


Figure S10. Comparison of BayesRR-RC results obtained with 5,000 versus 50,000 iterations in a simple scenario. The model was run on data from a simple scenario where OCR contributed to 50% of the genetic variance. The 5,000 iterations correspond to the values used in the present study (burn-in from iterations 1-2,000), while 50,000 iterations correspond to a longer run (burn-in from iterations 1-5,000). A) Estimated %SNP heritability per iteration. Iterations used for parameter estimation in the standard run are delimited by the two blue dashed lines located at iterations 2,001 and 5,000. B) Distribution of %SNP heritability estimates in iterations 2,001-5,000 (standard run) and 5,001-50,000 (long run). C) %SNP heritability estimates for 25 simulations estimated using BayesRR-RC with 5,000 versus 50,000 iterations.


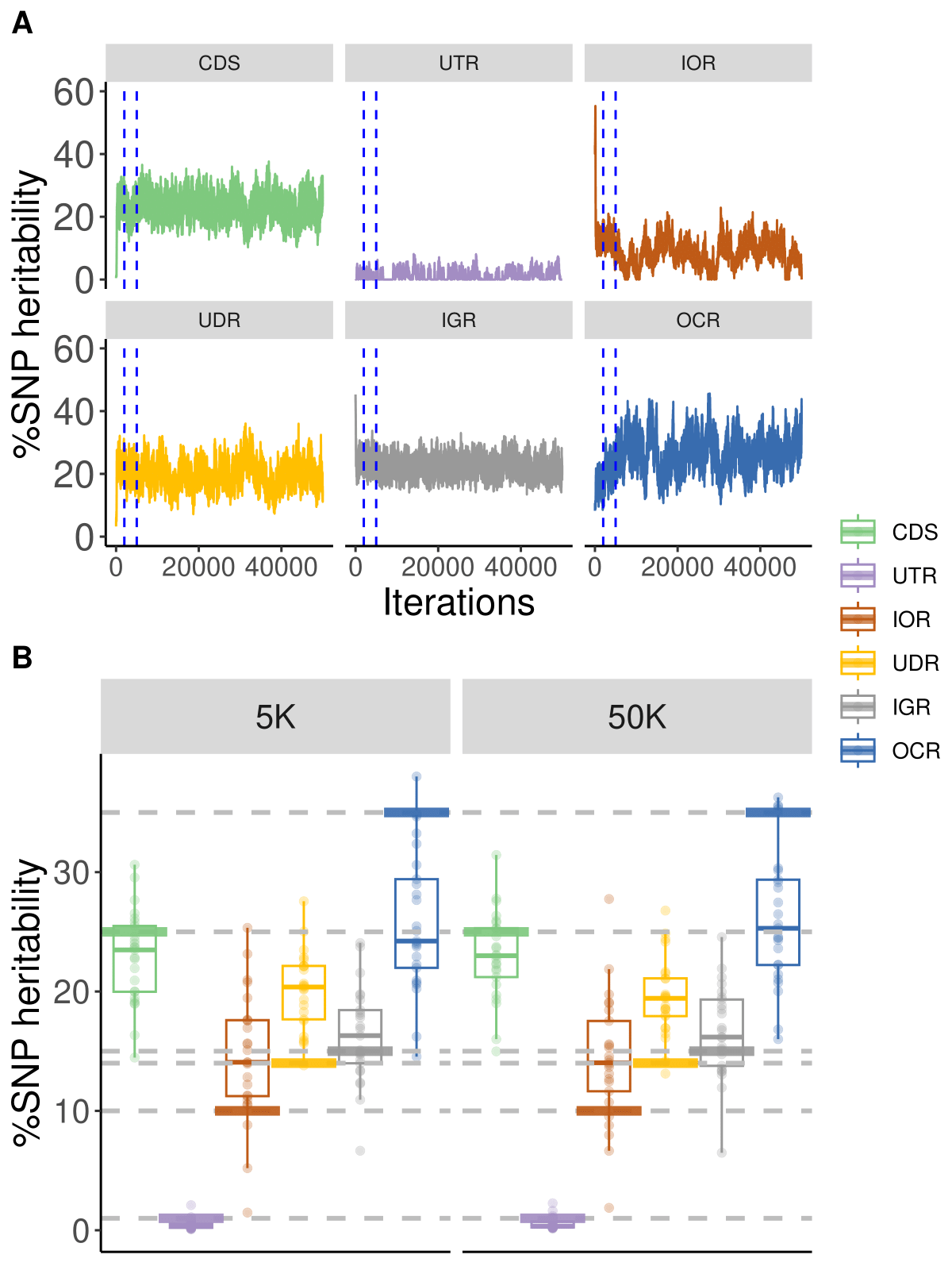


Figure S11. Comparison of BayesRR-RC results obtained with 5,000 versus 50,000 iterations in the first complex scenario. The 5,000 iterations correspond to the values used in the present study (burn-in from iterations 1-2,000), while 50,000 iterations correspond to a longer run (burn-in from iterations 1-5,000). A) Estimated %SNP heritability per iteration for the six components. Iterations used for parameter estimation in the standard run are delimited by the two blue dashed lines located at iterations 2,001 and 5,000. B) %SNP heritability estimates for the six components estimated using BayesRR-RC with 5,000 versus 50,000 iterations in 25 simulations.
